# ACCERBATIN, a small molecule at the intersection of auxin and reactive oxygen species homeostasis with herbicidal properties

**DOI:** 10.1093/jxb/erx242

**Published:** 2017-07-26

**Authors:** Yuming Hu, Thomas Depaepe, Dajo Smet, Klara Hoyerova, Petr Klíma, Ann Cuypers, Sean Cutler, Dieter Buyst, Kris Morreel, Wout Boerjan, José Martins, Jan Petrášek, Filip Vandenbussche, Dominique Van Der Straeten

**Affiliations:** 1 Laboratory of Functional Plant Biology, Department of Biology, Faculty of Sciences, Ghent University, K.L. Ledeganckstraat, Ghent, Belgium; 2 Institute of Experimental Botany ASCR, Praha, Czech Republic; 3 Centre for Environmental Sciences, Hasselt University, Agoralaan Building D, Diepenbeek, Belgium; 4 Department of Botany and Plant Sciences, Institute of Integrative Genome Biology, University of California, Riverside, CA, USA; 5 NMR and Structure Analysis, Department of Organic Chemistry, Krijgslaan, Ghent, Belgium; 6 Department of Plant Systems Biology, VIB (Flanders Institute for Biotechnology), Technologiepark, Ghent, Belgium

**Keywords:** Arabidopsis, auxin homeostasis, chemical genetics, ethylene signaling, herbicide, quinoline carboxamide, reactive oxygen species, triple response

## Abstract

The volatile two-carbon hormone ethylene acts in concert with an array of signals to affect etiolated seedling development. From a chemical screen, we isolated a quinoline carboxamide designated ACCERBATIN (AEX) that exacerbates the 1-aminocyclopropane-1-carboxylic acid-induced triple response, typical for ethylene-treated seedlings in darkness. Phenotypic analyses revealed distinct AEX effects including inhibition of root hair development and shortening of the root meristem. Mutant analysis and reporter studies further suggested that AEX most probably acts in parallel to ethylene signaling. We demonstrated that AEX functions at the intersection of auxin metabolism and reactive oxygen species (ROS) homeostasis. AEX inhibited auxin efflux in BY-2 cells and promoted indole-3-acetic acid (IAA) oxidation in the shoot apical meristem and cotyledons of etiolated seedlings. Gene expression studies and superoxide/hydrogen peroxide staining further revealed that the disrupted auxin homeostasis was accompanied by oxidative stress. Interestingly, in light conditions, AEX exhibited properties reminiscent of the quinoline carboxylate-type auxin-like herbicides. We propose that AEX interferes with auxin transport from its major biosynthesis sites, either as a direct consequence of poor basipetal transport from the shoot meristematic region, or indirectly, through excessive IAA oxidation and ROS accumulation. Further investigation of AEX can provide new insights into the mechanisms connecting auxin and ROS homeostasis in plant development and provide useful tools to study auxin-type herbicides.

## Teaser

ACCERBATIN is an ethylene-mimicking small molecule that affects auxin homeostasis and ROS accumulation in etiolated seedlings. In light-grown plants, it exhibits auxin-like herbicidal properties.

## Introduction

Ethylene is a gaseous plant hormone regulating many aspects of plant development and response to stress (Abeles *et al.*, 1992). Ethylene effects on dark-grown pea seedlings were described as the triple response (Neljubov, 1901; Knight *et al.*, 1910), and in Arabidopsis the triple response phenotype includes exaggerated curvature of the apical hook, reduced hypocotyl and root length, and increased radial expansion of the hypocotyl (Bleecker *et al.*, 1988).

Ethylene is synthesized by almost all plant tissues from methionine, via *S*-adenosylmethionine (AdoMet) and 1-aminocyclopropane-1-carboxylic acid (ACC) (Yang and Hoffman, 1984; Van de Poel and Van Der Straeten, 2014). ACC is oxidized to ethylene by ACC oxidase (ACO). Several *ethylene overproducing* mutants have been identified, namely *eto1*, *eto2*, and *eto3*, which fail to regulate ACC synthase (ACS) stability resulting in increased ethylene production (Chae *et al.*, 2003).

Ethylene signaling is initiated by inactivation of copper containing ethylene receptors, ETHYLENE RESPONSE 1 (ETR1), ETHYLENE RESPONSE SENSOR 1 (ERS1), ETHYLENE RESPONSE 2 (ETR2), ETHYLENE-INSENSITIVE 4 (EIN4), and ETHYLENE RESPONSE SENSOR 2 (ERS2), located at the endoplasmic reticulum (ER) membrane and Golgi apparatus (Dong *et al.*, 2010). Upon ethylene binding to its receptors, the CONSTITUTIVE TRIPLE RESPONSE (CTR1) kinase is inactivated, preventing phosphorylation of the EIN2 C-terminal domain, which results in its proteolytic cleavage and movement to the nucleus (Ju *et al.*, 2012). Subsequently, the EIN2 C-terminus activates the downstream transcriptional factors, EIN3 and EIN3-LIKE (EILs), which in turn switch on transcription of ETHYLENE RESPONSE FACTORs (ERFs) and ETHYLENE RESPONSE DNA BINDING FACTOR (EDFs) (Alonso *et al.*, 2003).

Many ethylene effects on growth and development of young seedlings in darkness are auxin-mediated and vice versa (Muday *et al.*, 2012). Ethylene and auxin act synergistically in root elongation and root hair formation (Masucci and Schiefelbein, 1994; Pitts *et al.*, 1998; Rahman *et al.*, 2002), while working antagonistically or independently in controlling hypocotyl elongation (Burg and Burg, 1966; Suttle, 1988; Collett *et al.*, 2000). Recent research on auxin–ethylene crosstalk in hypocotyl growth focused on apical hook development. Hook formation results from differential cell elongation (Raz and Ecker, 1999), driven by an auxin maximum at the concave side (Schwark and Schierle, 1992; Lehman *et al.*, 1996).

Exogenous auxins and polar auxin transport (PAT) inhibitors suppress hook curvature. Likewise, some mutants with defective auxin synthesis, transport or signaling display a hook-deficient or hookless phenotype (Harper *et al.*, 2000; Stepanova *et al.*, 2008; Vandenbussche *et al.*, 2010; Wu *et al.*, 2010; Zadnikova *et al.*, 2010). Further evidence for an auxin–ethylene interaction comes from *HOOKLESS1* (*HLS1*), the transcription of which can be activated through EIN3 (Lehman *et al.*, 1996; An *et al.*, 2012). HLS1 inhibits accumulation of AUXIN RESPONSE FACTOR2 (ARF2), a repressor controlling differential auxin responses (Li *et al.*, 2004).

Chemical genetics has led to the identification of new compounds to help in dissecting plant hormone pathways, e.g. bikinin (De Rybel *et al.*, 2009) and pyrabactin (Park *et al.*, 2009). Ethylene relevant chemicals include quinazolinone inhibitors of ACS (Lin *et al.*, 2010), L-kynurenine, an inhibitor of TRYPTOPHAN AMINOTRANSFERASE OF ARABIDOPSIS1/TAA RELATED (TAA1/TAR), key enzymes in ethylene-mediated auxin biosynthesis (He *et al.*, 2011), as well as brassinopride, an inhibitor of brassinosteroid action that also promotes ethylene response (Gendron *et al.*, 2008). In addition, the use of small molecules discovered in Arabidopsis can be translated to crop species (Schreiber *et al.*, 2011). We previously screened a 12 000 compound chemical library for molecules that altered the triple response phenotype triggered by ACC in etiolated Arabidopsis seedlings (Hu *et al.*, 2014). Here, we report follow-up work on the quinoline carboxamide compound ACCERBATIN (AEX), which was selected based on its exacerbation of the triple response.

## Materials and methods

### Plant material

Col-0, *eto2-1*, *etr1-1*, *arf2-6*, *nph4-1arf19-1*, *aux1-7*, 35S::PIN1, *rcn1-1*, *pgp4-1*, *abcb1abcb19*, and *axr3-1* were from the Nottingham Arabidopsis Stock Centre. *ctr1-1* and *hls1-1* were from Arabidopsis Biological Resource Center. *ein2-1*, *ein3-1eil1-1* and *EBS::GUS* 1–11 lines were a kind gift from J. Ecker (The Salk Institute, San Diego, USA). *pCYCB1;1::DB-GUS* was provided by L. De Veylder (Flemish Institute of Biotechnology, Ghent, Belgium). The *DR5::GUS* line was provided by T. Guilfoyle (University of Missouri, USA). *aux1lax3* and *aux1lax1lax2lax3* were from M. Bennett (University of Nottingham, UK). *pin3-3* was from O. Tietz (Albert-Ludwigs-Universität, Germany). *pid salk*, *wag1*, *wag2*, and *wag1wag2pid* were provided by Remko Offringa (Leiden University, the Netherlands). All lines were in Col-0 background.

### Growth conditions

Surface-sterilized seeds were sown on half-strength MS (Duchefa) medium (1% sucrose (pH5.7), 0.8% agar (LABM)). ACCERBATIN (AEX; ID: 6527749) and analogs 6640029, 6520852, and 6514196 were procured from ChemBridge (www.hit2lead.com, last accessed 16 July 2017). LAT014C06, LAT013C04, LAT007H11, LAT010G08, and LAT024E02 were selected from the LATCA library (www.thecutlerlab.org/2008/05/latca.html, last accessed 16 July 2017), originally obtained from ChemBridge (ID 5601004, 5707885, 5473152, 5617132, and 5712036, respectively). Stock solutions were prepared in dimethyl sulfoxide (DMSO; Sigma-Aldrich). 1-Aminocyclopropane-1-carboxylic acid (ACC; dissolved in deionized water), *N*-(1-naphtyl)phtalamic acid (NPA; in DMSO), 1-naphthoxyacetic acid (1-NOA; in DMSO), indole-3-acetic acid (IAA; in ethanol) and 2,4-dichlorophenoxyacetic acid (2,4-D; in ethanol) were from Sigma-Aldrich. DMSO was supplied in the same final concentration in all treatments. For assays in darkness, seeds were stratified at 4 °C for 2 d, exposed to light for 6 h to stimulate germination, and returned to darkness (22 °C) for the desired time. For assays in light, plates were transferred to a tissue culture chamber (22 °C; 16 h/8 h light/dark) after stratification. Ethylene exposure was performed by flushing plants continuously with 5 ppm ethylene in air (Air Liquide) in a sealed 5-liter jar. A control was performed with ethylene-free air. For the foliar spraying experiments, plantlets were grown on jiffy pellets and left untreated, or sprayed either with 100 µM AEX (containing 0.1–0.2% v/v Tween 20 (Sigma-Aldrich) in diH_2_O) or with 0.1–0.2% v/v Tween 20 until leaves were wet.

### Quantification of phenotypes

The length and angle were measured by ImageJ (National Institutes of Health). The angle of hook curvature was measured as defined previously (Vandenbussche *et al.*, 2010). The number of cells along the apical–basal axis of the hypocotyl were obtained by counting a cortex cell file at the outer side of the hook. The apical hook region was defined starting from the first cell at the bifurcation of the vascular bundle below the cotyledons until the first obviously elongated cell. The number of cells in the root meristematic zone was obtained by counting cells showing no signs of rapid elongation within a cortex cell file (Beemster and Baskin, 1998). Patterning of the root developmental zones was based on Verbelen *et al.* (2006). Rosette area was measured with the ImageJ plug-in Rosette tracker (De Vylder *et al.*, 2012). For close-ups, seedlings were mounted on a microscope slide in a chloral hydrate (Acros, Geel, Belgium) solution and viewed with a Zeiss Axiovert 200 microscope (×20 Plan Apochromat objective). For kinetic analysis of apical hook development, time-lapse images were taken in the dark using an infrared imaging system (Smet *et al.*, 2014).

### Liquid chromatography–mass spectrometry profiling

All samples were profiled via reversed phase ultrahigh performance liquid chromatography (RP-UHPLC) connected to a Fourier transform–ion cyclotron resonance mass spectrometer (FT-ICR-MS) as previously published (Morreel *et al.*, 2014). Modifications included the column type (Acquity UPLC BEH C18; 150 mm × 2.1 mm, 1.8 μm; Waters, Milford, MA, USA) and the use of atmospheric pressure chemical ionization (APCI). Here, a gradient from 95% aqueous formic acid to 100% acetonitrile was performed in 35 min at a column temperature of 80 °C. The APCI source was operated using 3.5 μA, 200 °C, 300 °C, 40 arbitrary units (arb) and 20 arb for the source current, capillary temperature, vaporizer temperature, sheath gas and auxiliary gas flow rates, respectively. Full MS spectra in the range *m*/*z* 120–650 were recorded in the negative ionization mode.

### Nuclear magnetic resonance spectrometry

All nuclear magnetic resonance (NMR) spectra were measured on an Avance II Bruker spectrometer operating at a ^1^H frequency of 500 MHz and equipped with a ^1^H/^13^C/^31^P TXI-z probe. Three samples were provided, each containing 0.5 mg of product dissolved in 53 µl of protonated methanol and further diluted to 600 µl total volume using deuterated methanol. One standard and two samples heated at 50 °C for 30 min and 80 °C for 1 hour respectively, were analysed. All spectra were referenced to the protonated methyl solvent signal at 3.34 (1) ppm for the ^1^H frequency. The experiments recorded on the samples included 1D ^1^H spectra recorded at room temperature for each sample provided. In addition, a temperature stability study was performed with spectra recorded at regular intervals (30 min) at 50 °C over a period of 12 h. Finally, a small-scale pH stability study was performed where both the original reference sample and the sample which was heated at 80 °C were measured at two different pH values (pH 4 and pH 5). All spectra were processed using TOPSPIN 3.2 pl3 software (http://www.bruker.com/products/mr/nmr/nmr-software/software/topspin/overview.html, last accessed 16 July 2017).

### Histochemical staining

For glucuronidase assays, seedlings of β-glucuronidase (GUS) reporter lines were treated with 90% ice-cold acetone, washed with 0.1 M phosphate buffer (pH 7.2) and incubated at 37 °C overnight in GUS buffer (2 mM 5-bromo-4-chloro-3-indolyl-glucuronide (X-gluc; Duchefa, The Netherlands)). To detect the accumulation of reactive oxygen species, seedlings were stained with diaminobenzidine (DAB) for hydrogen peroxide and with nitroblue tetrazolium (NBT) for superoxide, essentially performed according to He *et al.*, (2012). 3,3′-Diaminobenzidine tetrahydrochloride dihydrate (DAB; Sigma-Aldrich) at 1 mg ml^−1^ was prepared in diH_2_O, and adjusted to pH 3.8 with Tris–HCl buffer (pH 7.5). For the NBT (Sigma-Aldrich) staining, 2 mM NBT solution was prepared in 20 mM phosphate buffer (pH 6.1). Incubation in DAB solution was for 8 h and incubation in NBT solution was for 3 h, in darkness. Seedlings were kept in 70% ethanol for further differential interference contrast (DIC) microscopy analysis.

### Measurement of ethylene emanation

Ethylene emanation was measured with a photo-acoustic detector (ETD-300 ethylene detector, Sensor Sense, The Netherlands) and was essentially performed as described in Ellison *et al.* (2011).

### Determination of the effects of AEX on gravitropism

The gravitropism assay was performed as described previously (Vandenbussche *et al.*, 2011) with reorientation of 3-day-old seedlings and subsequent analysis after 24 h.

### Auxin accumulation assays in tobacco BY-2 suspension cells

Tobacco BY-2 cells (*Nicotiana tabacum* L., cv. Bright Yellow-2; Nagata *et al.*, 1992) were cultivated as described previously (Petrášek *et al.*, 2003). Auxin efflux was measured by cellular changes in accumulation of radioactive 1-naphthaleneacetic acid (NAA) ([^3^H]NAA) (Petrášek and Zažímalová, 2006). The accumulation of 2 nM [^3^H]NAA (American Radiolabeled Chemicals, Inc.) in cells treated with AEX or ACC was determined by liquid scintillation counting (Packard Tri-Carb 2900TR scintillation counter; PerkinElmer). Cell surface radioactivity was corrected by subtracting counts of aliquots collected immediately after addition of [^3^H]NAA. Counts were converted to pmol of [^3^H]NAA per 1 million cells.

### Determination of endogenous auxin and auxin metabolites

Cotyledons (with shoot apical meristems) and hypocotyls (60–80 pieces) of 4-day-old Arabidopsis seedlings grown in darkness were separated in darkness. Pieces were collected in 300 ml methanol. The cutting positions are illustrated in Supplementary Fig. S1 at *JXB* online. After overnight extraction at –20 °C, tissue debris was separated by centrifugation (10 000 *g*) and extracts were evaporated to dryness. Quantification of auxin and auxin metabolites was performed according to Dobrev and Vankova (2012).

### Global gene expression analysis

Eight hundred seeds were sterilized with chlorine gas and subsequently grown in darkness on agar-supplemented medium. After 60 h, seedlings were transferred to liquid half-strength MS medium supplemented with AEX (100 µM) or with an equivalent volume of DMSO as a control and treated for 6 h. Three independent experiments were performed. RNA isolation was done using an RNeasy Mini Kit (Qiagen). For each sample, more than 1 µg RNA was sent to the Affy Gene Chip Service (NASC) for analysis on the Arabidopsis ATH1 Genome Array (Affymetrix). Quality assessment, normalization and statistical analysis of microarray data were done with Robin software (Lohse *et al.*, 2010). The Robust Multichip Average (RMA) algorithm was applied to create an expression matrix (Irizarry *et al.*, 2003), and the false discovery rate (FDR) was chosen for *P*-value correction (Benjamini *et al.*, 2001). The significance cut-off was defined as a log2-fold change in expression less than 1 and genes showing a *P*-value greater than 0.05 were chosen. Gene annotation search was done in TAIR. The overrepresentation of Gene Ontology groups on sets of differentially expressed genes was studied with BiNGO software (Maere *et al.*, 2005). For auxin-related genes, data from Okushima *et al.* (2005*a*) were derived from the Gene Expression Omnibus (GEO) database (http://www.ncbi.nlm.nih.gov/geo/, last accessed 16 July 2017) with accession number GSE627 (samples GSM9620 and GSM9624 to GSM9628); for ethylene-related genes, data from Olmedo *et al.* (2006) with accession number GSE5174 (samples GSM116733 to GSM116736) were used.

### *In silico* docking of AEX in TIR1

Docking analysis was performed in Autodock/Vina (Trott & Olson, 2010), using the crystal structure of TIR1-ASK1-IAA7 (Tan *et al.*, 2007; PDB ID:29Q1) and simulation parameters according to Hayashi *et al.* (2008). Visualization of the TIR1 binding cavity was done in PyMOL (Schrödinger Inc., New York, USA).

### Accession numbers

Sequence data from this article can be found in the EMBL/GenBank data libraries under accession numbers AT1G19020, AT1G05340, AT2G21640, AT1G57630, PRP3 (AT3G62680), LRX1 (AT1G12040), EXPA7 (AT1G12560), EXPA18 (AT1G62980) and UPB1 (AT2G47270).

### Statistics

Quantitative data are presented as means±SD. Statistical analysis was performed in R 3.2.3. (R Foundation for Statistical Computing, Vienna, Austria; https://www.R-project.org/, last accessed 16 July 2017). Comparison of means among three or more groups was done with analysis of variance. Normality of the residuals and homoscedasticity were verified with quantile–quantile plots and boxplots, respectively. Due to violation of these assumptions, non-parametric alternatives were chosen. The Kruskal–Wallis rank sum test was applied in the case of one categoric variable; the Scheirer–Ray–Hare extension was applied for two categoric variables. The *post hoc* Wilcoxon’s rank sum test (*P*<0.05) was performed for multiple pairwise comparisons (with the Bonferroni correction). Wilcoxon’s rank sum test was also applied to test for differences between the distributions of only two groups. Output of the statistical analyses can be found in Supplementary Table S1.

## Results

### Identification of ACCERBATIN, a compound exacerbating the triple response

**Fig. 1.**
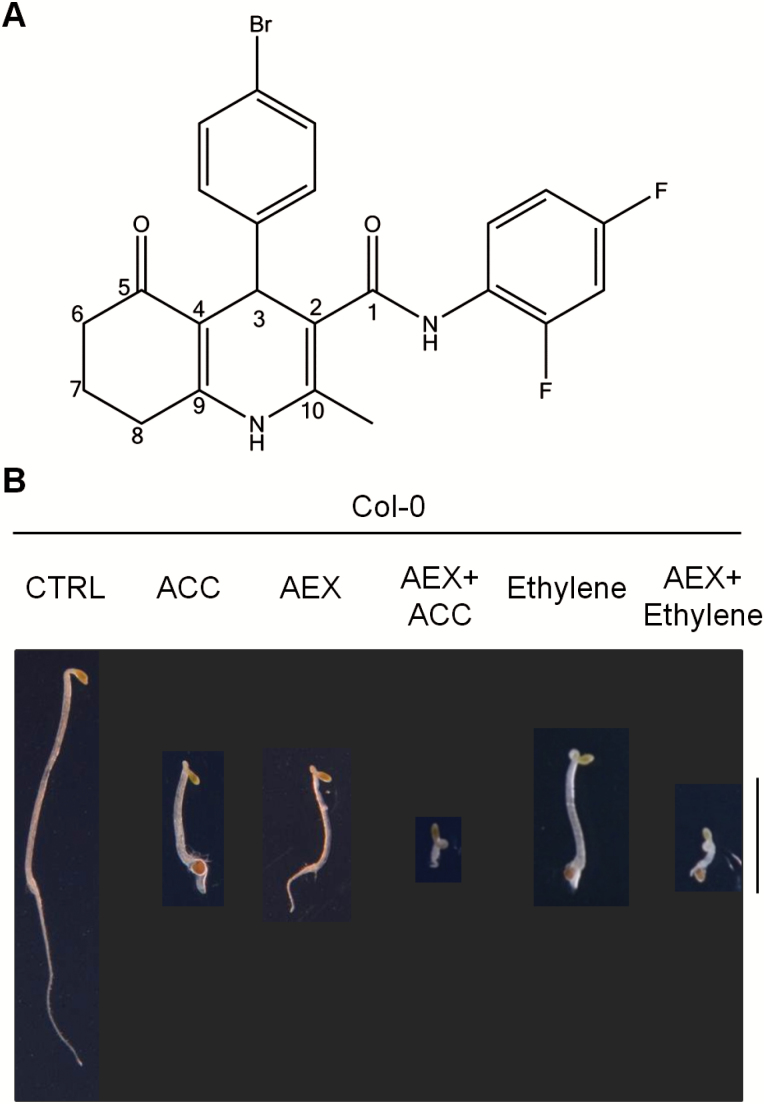
AEX enhances the triple response phenotype in Arabidopsis. (A) Chemical structure of AEX. IUPAC name: 4-(4-bromophenyl)-*N*-(2,4-difluorophenyl)-2-methyl-5-oxo-1,4,5,6,7,8-hexahydro-3-quinolinecarboxamide. Chembridge ID: 6527749. The carbon atoms of the quinoline carboxamide core are numbered. (B) Four-day etiolated seedlings of wild-type (Col-0) were grown on horizontal plates using half-strength MS medium containing 1% sucrose supplemented with 0.05% DMSO (CTRL), 10 µM ACC, 50 µM AEX, 10 µM ACC+50 µM AEX, or placed in air supplied with 5 ppm of ethylene or treated with the combination of 50 µM AEX+5 ppm ethylene. All treatments contained 0.05% DMSO. Individual photographs were cropped without changing the scale; the black background was post-added. Scale bar: 5 mm. (This figure is available in color at *JXB* online.)

Recently, a series of chemicals altering the ACC-induced triple response phenotype of etiolated Arabidopsis seedlings were identified from a high-throughput chemical genetics screen (Hu *et al.*, 2014). A quinoline carboxamide compound, called ACCERBATIN (AEX), was chosen for further investigation (Fig. 1A). Four-day-old etiolated seedlings treated with AEX displayed a phenotype mimicking the triple response, including an exaggerated apical hook, as well as shortening of the hypocotyl and the root, but without conspicuous lateral expansion of the hypocotyl (Fig. 1B). Combined treatment with 50 µM AEX and either 10 µM ACC or 5 ppm ethylene enhanced the effect of ethylene or its precursor. The exacerbated triple response phenotype was characterized by an even stronger apical hook curvature, and a more severe shortening of both the hypocotyl and the root (Fig. 1B).

**Fig. 2.**
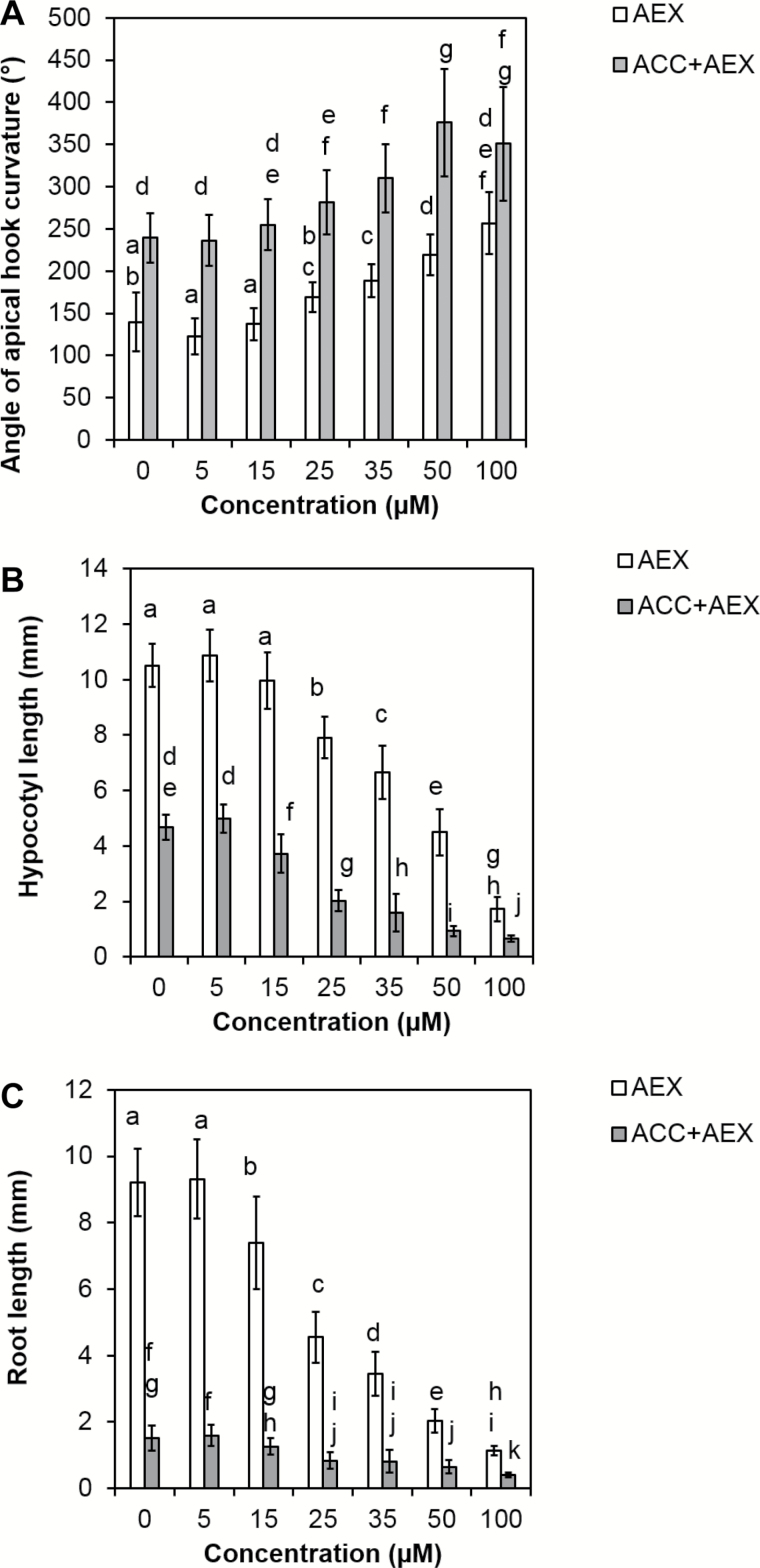
Dose–response of 4-day-old etiolated seedlings exposed to AEX concentrations ranging from 0 to 100 µM, grown on horizontal plates. The apical hook angle (A), hypocotyl (B) and root (C) lengths were measured. White bars represent dose–response effects of AEX alone (at 0 µM AEX, growth medium is supplied with 0.05% DMSO); grey bars represent AEX dose–response effects in the presence of 10 µM ACC. Data are presented as means±SD. Statistical analysis was performed by means of the Scheirer–Ray–Hare extension of the non-parametric Kruskal–Wallis rank sum test. The dependent variables (rank-transformed) apical hook curvature (11>*n*>30; A), hypocotyl length (10>*n*>52; B) and root length (10>*n*>52; C) were compared among treatments and different concentrations (categorical variables). Multiple pairwise comparisons were performed with *post hoc* Wilcoxon’s rank sum tests (*P*<0.05) and *P*-values were adjusted with the Bonferroni correction. Bars with at least one letter in common are not significantly different.

In order to determine the minimal concentration at which AEX affects seedling growth, a dose–response assay was performed. Fifty micromolar AEX was necessary to quantitatively mimic the apical hook exaggeration and inhibition of root and hypocotyl elongation induced by 10 µM ACC (Fig. 2A–C). In combination with 10 µM ACC, the effects of AEX on apical hook development, hypocotyl and root were additive in all concentrations tested. Based on the above-mentioned findings, 50 µM of AEX was mostly used for further investigations to explore its function.

### AEX stability *in planta*

Many chemicals act *in planta* indirectly, i.e. through the action of a breakdown product (e.g. pro-auxins, Savaldi-Goldstein *et al.*, 2008). Therefore, we assessed whether AEX can be metabolized. Liquid chromatography–mass spectrometry (LC-MS) spectra of etiolated AEX-treated seedlings, which were continuously treated for 4 d or only 6 h on day 3, revealed the presence of intact AEX (Supplementary Protocol S1). In addition, a compound with chemical formula C_19_H_17_O_2_NBr was found, corresponding to the loss of a C_4_H_2_NF_2_ fragment from AEX (C_23_H_19_O_2_N_2_BrF_2_), possibly formed by cleavage of the amide bond followed by addition of an ethyn moiety, since the amide cleavage would have resulted in the loss of six carbons and four hydrogens (Supplementary Protocol S1). To assess temperature and pH stability, AEX was analysed by ^1^H-NMR spectroscopy after heating (up to 80 °C) or acid treatment (pH 4) by HCl. Neither one of these experiments revealed notable differences, leading to the conclusion that AEX is both thermally stable and pH stable *in vitro* (see Supplementary Protocol S2 and Supplementary Fig. S2).

### Effects of AEX on the shoot: hypocotyl growth and apical hook curvature

**Fig. 3.**
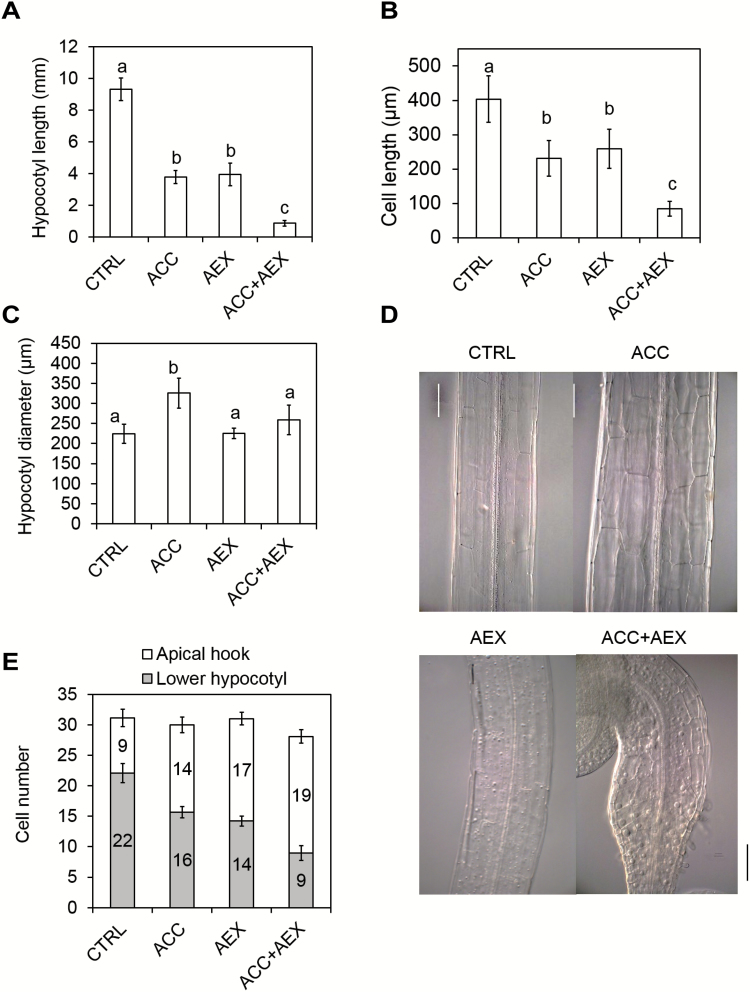
Effect of AEX on apical hook and hypocotyl growth of 4-day-old etiolated Col-0 seedlings, grown on horizontal plates. (A–C) Length (A) and diameter (C) (of the middle part) of hypocotyls and the corresponding length (B) of cortex cells from the middle part of the hypocotyl were measured. (D) DIC images of the middle part of the hypocotyl of 4-day-old etiolated seedlings. From left to right and top to bottom: CTRL, ACC, AEX, AEX+ACC. (E) Numbers of cortex cells along the convex side of the apical hook and the lower part of the hypocotyl. Data are presented as means±SD. Statistical analysis was performed by means of a Kruskal–Wallis rank sum test. Multiple pairwise comparisons were performed with *post hoc* Wilcoxon’s rank sum tests (*P*<0.05); *P*-values were corrected with the Bonferroni correction. Bars with at least one letter in common are not significantly different. Scale bar: 100 µm. (This figure is available in color at *JXB* online.)

To investigate how AEX affects hypocotyl growth at the cellular level, cortex cell dimensions were quantified (Fig. 3). Fifty micromolar AEX alone inhibited hypocotyl elongation of 4-day-old etiolated seedlings compared with control seedlings, while combining AEX and ACC had an additive inhibitory effect (Fig. 3A). These data were largely supported by a significant decrease in cortex cell length for AEX and ACC (Fig. 3B, D). However, in contrast to 10 µM ACC, which increased radial expansion by 1.5-fold compared with the control, AEX alone did not significantly alter the hypocotyl diameter. In combination with ACC, hypocotyl diameter was weakly increased compared with control indicating a negative effect of AEX on ACC-mediated lateral expansion (Fig. 3C, D).

Apical hook formation in etiolated seedlings is dependent on cell division and differential elongation of hypocotyl cells (Raz and Ecker, 1999; Raz and Koornneef, 2001). The *pCYCB1;1::DB-GUS* construct with a destruction box (DB) was used as a marker for cell division, indicating the number of cells in G2–M transition (Doerner *et al.*, 1996; Suzumori *et al.*, 2003). This number was significantly enhanced in the apical hook in the presence of AEX compared with the control (see Supplementary Fig. S3A). The total number of cells along the cortex cell file at the convex side of the apical hook and the basal portion of the hypocotyl were identical in AEX-treated and in control seedlings (31 cells) (Fig. 3E). However, the cortex cell distribution in the apical *versus* basal part of the hypocotyl differed between AEX-treated and control seedlings. Upon AEX treatment there were more cortex cells in the apical region (17 cells) as compared with the lower hypocotyl (14 cells) (number of cells in the apical hook divided by the number of cells in the lower hypocotyl=1.2), while the distribution in control seedlings was the opposite (ratio=0.4). Upon ACC treatment, there were fewer cortex cells in the apical hook compared with the lower hypocotyl, but the ratio was enhanced to 0.9 compared with the control. An additive effect was observed upon the combination of AEX and ACC (ratio=2). This differential cell distribution along the shoot indicated that AEX might affect cell fate within the hypocotyl.

### Effects of AEX on root growth

**Fig. 4.**
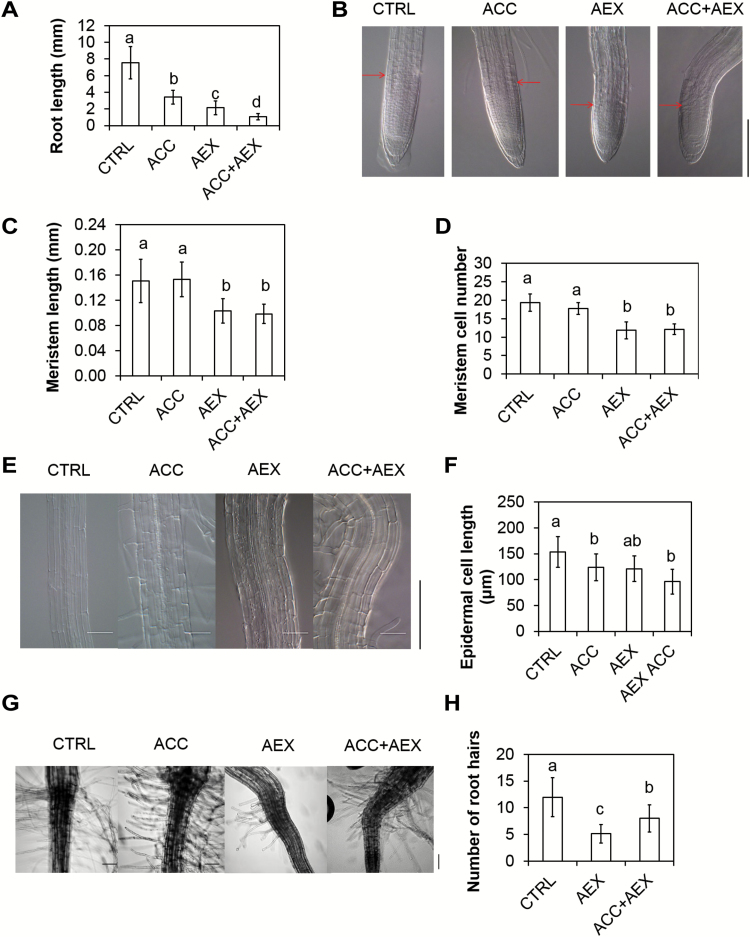
Inhibitory effect of AEX on root growth of 4-day-old etiolated Col-0 seedlings grown on vertically standing plates. (A) Root length. (B) DIC microscopy images of the root tip. The last cortical cells of the root meristem are marked with an arrow. Individual photographs were cropped without changing the scale. (C) Meristem length. (D) Meristem cell number. (E) DIC microscopy images of the root elongation zone. (F) Epidermal cell length in the root elongation zone. (G) Bright-field microscopy images of roots near the hypocotyl–root junction. (H) Total number of root hairs from one epidermal cell file. Data are presented as means±SD. Statistical analysis was performed by means of a Kruskal–Wallis test with root length (13>*n*>44; A), meristem length (13>*n*>17; C), meristem cell number (18>*n*>23; D), epidermal cell length (9>*n*>15; F) or number of root hairs (15>*n*>23; H) compared among treatments. Multiple pairwise comparisons were performed with *post hoc* Wilcoxon’s rank sum tests (*P*<0.05); *P*-values were adjusted with the Bonferroni correction. Bars with at least one letter in common are not significantly different. All experiments were performed at least twice and results from a representative experiment are shown. Scale bars: 100 µm. (This figure is available in color at *JXB* online.)

Root growth depends both on cell division rates in the root meristem and on longitudinal cell expansion in the elongation zone. Thus, the effects of AEX on primary root length, meristem size and activity, as well as epidermal cell length, were investigated. Seedlings grown on 50 µM AEX displayed a more severe reduction of root elongation as compared with those grown on 10 µM of ACC (Fig. 4A), while being even more pronounced on the combination of AEX and ACC. In contrast to the reduction upon ACC treatment, the inhibition of root length induced by AEX correlated with a shortening of the root meristem (Fig. 4B, C). Combining AEX and ACC had an additive effect on root shortening as compared with AEX alone, but the root meristem length was comparable to that of AEX-treated seedlings. Furthermore, cortex cell number was significantly reduced by AEX, either alone or combined with ACC, suggesting a suppressive effect on mitotic activity of root meristem cells (Fig. 4D). The latter was supported by a reduced expression of cell cycle marker *pCYCB1;1::DB-GUS* (see Supplementary Fig. S3A). ACC alone did not affect cell cycle activity, supporting a differential action of ACC and AEX on root elongation. In addition, AEX restricted elongation of epidermal cells that leave the root meristem although the extent of inhibition varied among seedlings (Fig. 4E, F), an effect that was also observed upon ACC or ethylene treatment (Le *et al.*, 2001; Růžička *et al.*, 2007). Altogether, these results indicate that AEX inhibits both cell division and elongation, as manifested by root shortening.

Another key feature in root growth related to ethylene/ACC response is root hair emergence and elongation (Tanimoto *et al.*, 1995). Both root hair length and number were negatively affected by AEX as compared with the control, while ACC exhibited a positive effect and combined treatment resulted in an intermediate effect (Fig. 4G, H). Thus, AEX represses both the ethylene-mediated root hair emergence and growth.

### AEX effects on ethylene biosynthesis and signaling

**Fig. 5.**
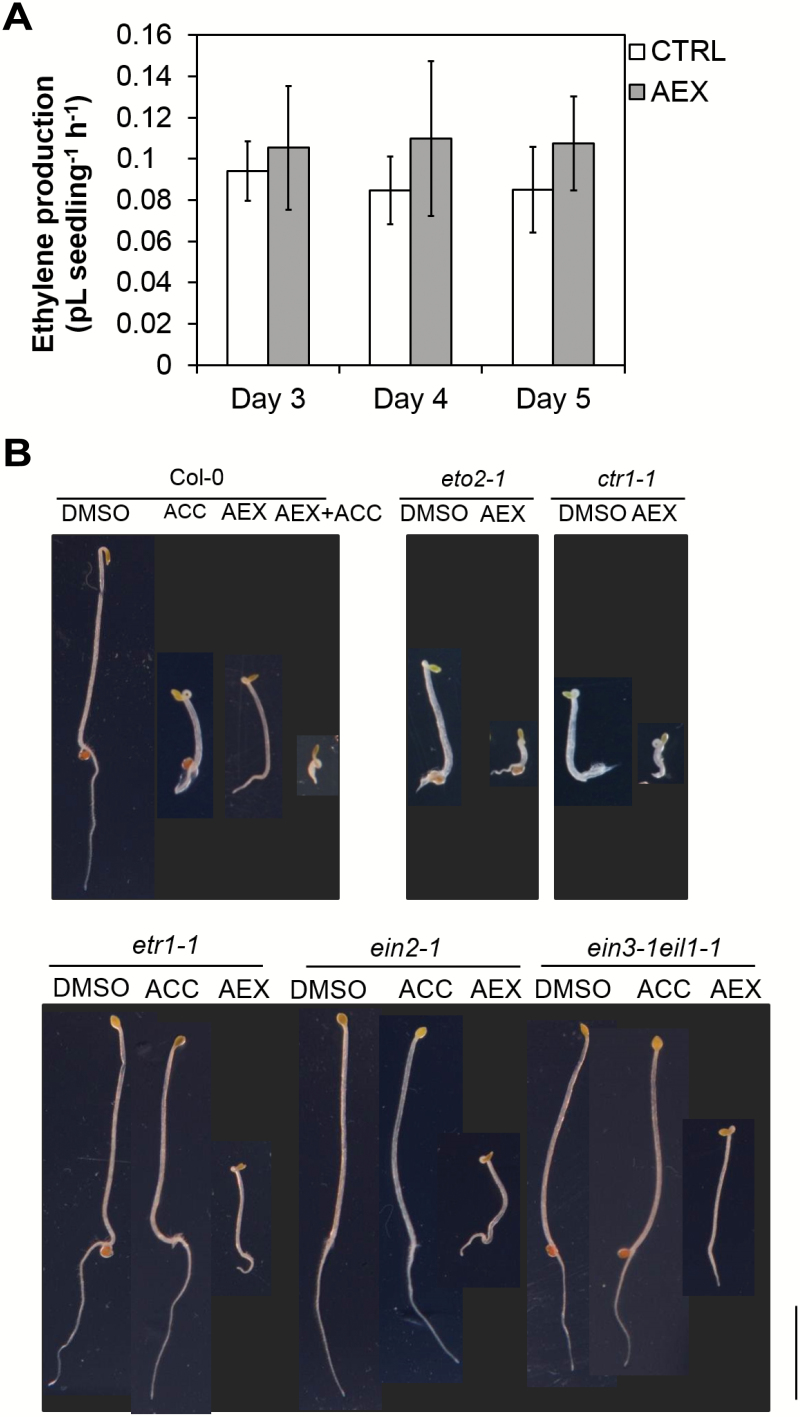
AEX effects on ethylene production and ethylene mutants. (A) Ethylene production of etiolated Col-0 seedlings in the presence of 50 µM AEX was not affected as compared with 0.05% DMSO control. Data are presented as means±SD. The experiments were performed twice with three replicates per condition with highly similar results; results of a representative experiment are shown. Statistical analysis did not detect significant differences between AEX and CTRL. (B) Four-day-old etiolated seedlings of Col-0, *eto2-1*, *etr1-1*, *ein2-1*, and *ein3-1eil1-1* grown on medium supplemented with 0.05% DMSO (CTRL), 10 µM ACC, 50 µM AEX, or 10 µM ACC+50 µM AEX (horizontal plates). All treatments contained 0.05% DMSO. The individual photographs were cropped without changing the scale; the black background was post-added. Scale bar: 5 mm. (This figure is available in color at *JXB* online.)

To determine whether the effect of AEX is dependent on ethylene biosynthesis, ethylene emanation of etiolated AEX-treated Col-0 seedlings was measured, using laser photo-acoustic spectroscopy. No significant effect was registered, indicating that, on growth, AEX most probably acts independent of ethylene biosynthesis (Fig. 5A).

To further dissect a possible site of action of AEX, a series of ethylene-related mutants were tested (Fig. 5B). Mutants exhibiting a constitutive ethylene response phenotype (*eto2-1* and *ctr1-1*) showed an exacerbated triple response phenotype in the presence of AEX. Interestingly, AEX triggered an enhanced apical hook curvature in the ethylene insensitive mutants *etr1-1*, *ein2-1*, and *ein3-1eil1-1* double mutant, while inhibiting elongation of both hypocotyl and root. Altogether, these data suggest that AEX acts downstream or, more likely, independent of ethylene signaling. The latter was strengthened by the fact that some phenotypes of AEX-treated seedlings are distinct from those typically observed in ACC-treated seedlings, as the absence of lateral expansion of the hypocotyl and the reduction of root hairs (*vide supra*). Moreover, AEX did not enhance expression of the ethylene reporter *EBS::GUS* (*GUS* gene driven by a synthetic *EIN3*-responsive promoter) (Stepanova *et al.*, 2007) in either shoots or roots, compared with control seedlings (see Supplementary Fig. S3B). In conclusion, the action of AEX most probably occurs in parallel to ethylene signaling.

### Effects of AEX on auxin responsiveness in shoot and root

Since etiolated seedling growth depends on auxin, *DR5::GUS* (Ulmasov *et al.*, 1997) expression was visualized (see Supplementary Fig. S3C). The auxin maximum appeared at the concave side of the hook in all conditions. However, when combining AEX with ACC, more cells were stained at the concave side and toward the basal end of the hypocotyl, rather than being restricted to the hook as in seedlings treated with ACC alone. This result confirmed that the effect of AEX on the apical hook is probably parallel to ethylene signaling, and is auxin-dependent. Kinematic (Smet *et al.*, 2014) and genetic analysis of the effect of AEX on hook development further supported these findings (Supplementary Figs S4 and S5, and Supplementary Protocol S3). In root tips, *DR5::GUS* was expressed in the quiescent center and columella in both AEX and control roots, while ACC expanded the area of staining, particularly in the vascular tissue. Remarkably, combining AEX with ACC reduced the signal compared with ACC alone, which was opposite to the effect seen in the apical hook.

### AEX enhances shoot gravitropism in darkness

**Fig. 6.**
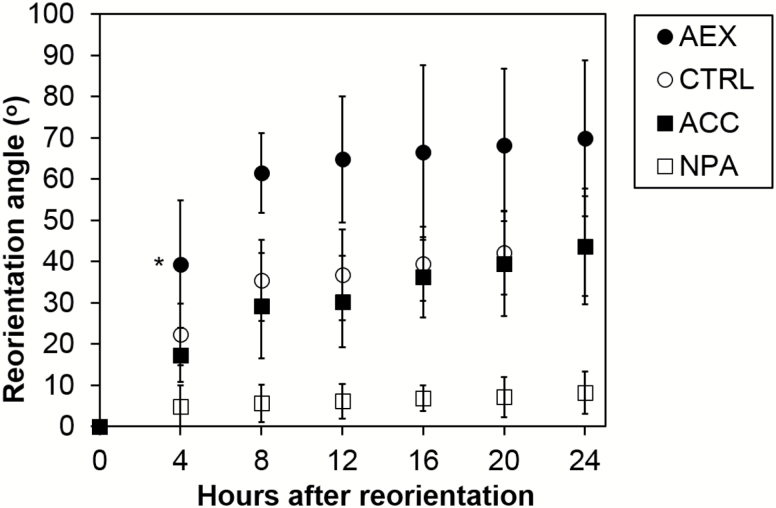
Response of etiolated Col-0 seedlings after growth and reorientation on vertically standing plates. Seedlings were grown in the presence of 0.05% DMSO (CTRL), 10 µM ACC, 50 µM AEX or 10 µM NPA. All treatments contained 0.05% DMSO. On day 2 after germination, plates were rotated by 90°, plants laying close to the horizontal axis were analysed, and the average reorientation angle of the hypocotyl was calculated. Ninety degrees corresponds with the new direction of the gravity vector. Data are presented as means±SD. Reorientation angle of AEX-treated seedlings at time point 4 h was compared with CTRL seedlings by means of Wilcoxon’s rank sum test (*P<0.05; 8>*n*>10). Experiments were performed twice with highly similar results; results of a representative experiment are shown. Results of a reorientation assay on selected mutants are shown in Supplementary Fig. S6.

Given the common mechanisms of differential growth in hook development and gravitropism (Zadnikova *et al.*, 2015), the effect of AEX on shoot gravitropism was determined by a reorientation assay (Nakamoto *et al.*, 2006). Consistent with previous reports (Nagashima *et al.*, 2008; Vandenbussche *et al.*, 2013) control seedlings and seedlings treated with ACC showed similar kinetics, and reoriented to an angle of 45° after 24 h, while NPA-treated seedlings did not react (Fig. 6). By contrast, AEX enhanced the rate of reorientation of wild-type (WT) seedlings significantly compared with control at as early as 4 h, reaching an angle of 70° after 24 h. Proper auxin signaling contributed to the stimulatory effects of AEX on asymmetric elongation in gravistimulated hypocotyls as the rate of reorientation in *msg2-1* (mutant in *IAA19*) and *nph4-1arf19-1* (carrying mutations in *ARF7* and *ARF19*) was not enhanced upon AEX (see Supplementary Fig. S6).

### AEX limits movement of free IAA produced from the shoot apical meristem and enhances auxin catabolism

**Fig. 7.**
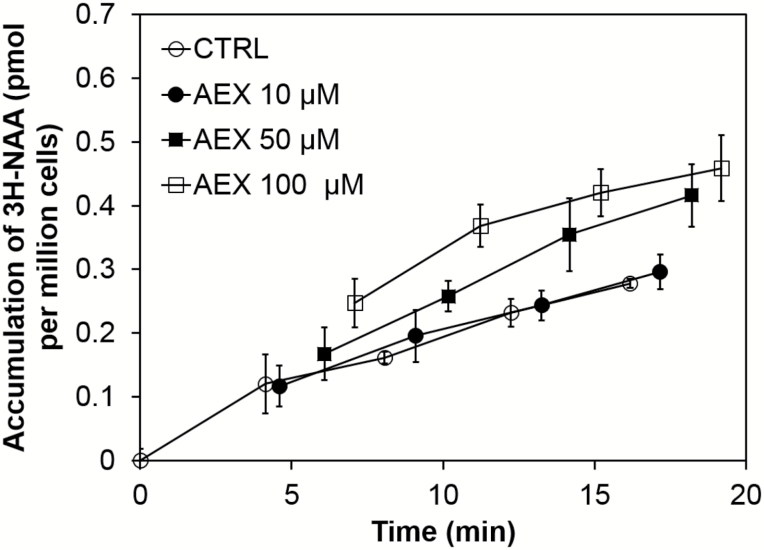
AEX inhibits auxin efflux in BY-2 cells. Radioactively labeled 1-naphthaleneacetic acid ([^3^H]NAA) accumulation kinetics in *Nicotiana tabacum* L., cv. Bright Yellow-2 cells upon treatment with 10, 50 and 100 µM AEX. Error bars indicate SD (*n*=4).

Since altered hypocotyl gravitropic response and apical hook formation result from asymmetric auxin distribution, which largely depends on altered auxin transport (Muday *et al.*, 2006; Vandenbussche *et al.*, 2010; Zadnikova *et al.*, 2010; Rakusova *et al.*, 2011), we aimed to verify whether AEX affects the auxin transport machinery. Auxin efflux was measured by cellular changes in accumulation of radioactively labeled NAA ([^3^H]NAA) in tobacco Bright Yellow (BY)-2 cells. NAA is a good substrate for active efflux but a weak substrate for active influx because it enters cells easily by diffusion (Delbarre *et al.*, 1996). An AEX dose-dependent effect was reflected in [^3^H]NAA accumulation kinetics, indicating inhibitory effects on auxin efflux (Fig. 7). The effective concentration (50 µM) fits to AEX dose-dependent effects for triple response-like phenotypes. Interestingly, simultaneous application of 100 µM ACC had no additive effect combined with 100 µM AEX, even though 100 µM ACC alone raised the accumulation slightly (see Supplementary Fig. S7).

**Fig. 8.**
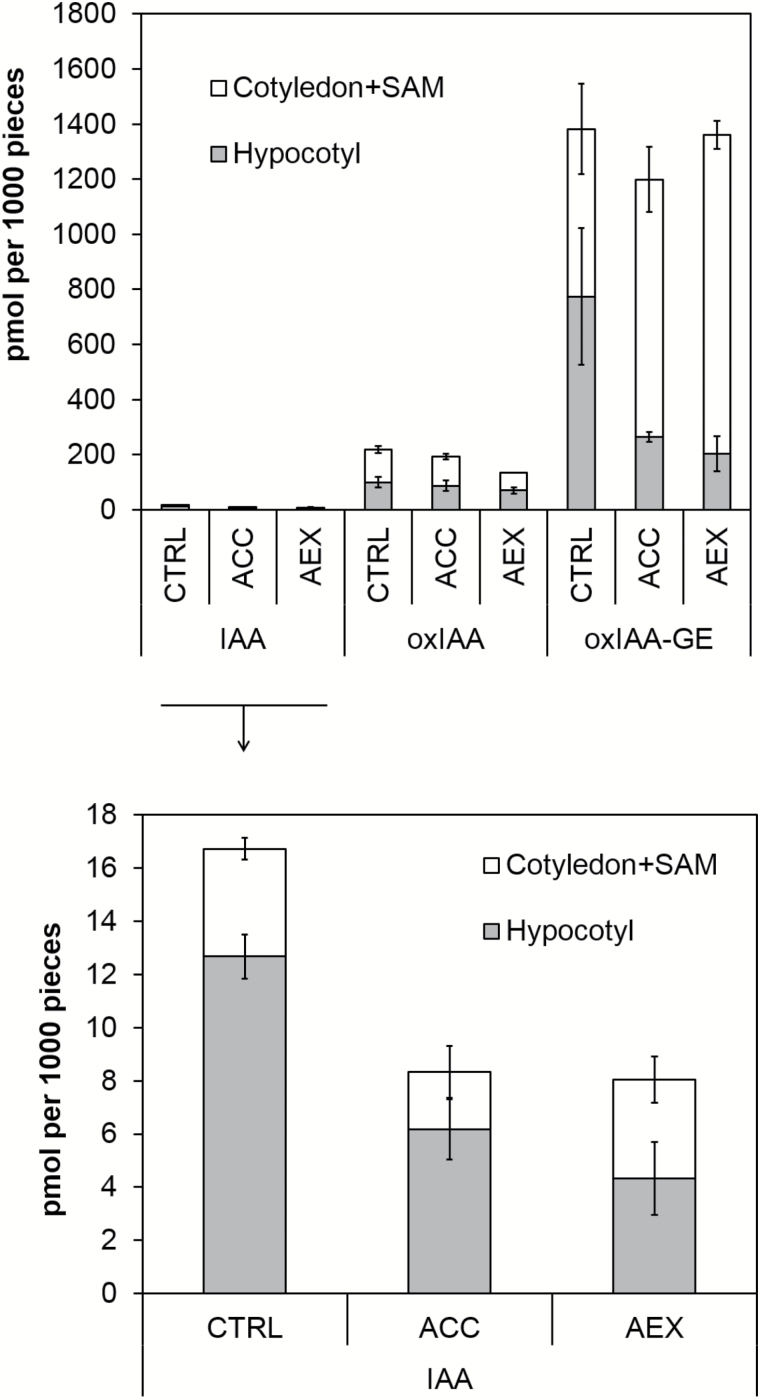
Endogenous auxin content in apices (including cotyledons and shoot apical meristems; SAMs) and hypocotyls treated with AEX. IAA, 2-oxindole-3-acetic acid (oxIAA) and oxIAA-glucose ester (oxIAA-GE) contents are shown for Col-0 treated with 0.05% DMSO (CTRL), 10 µM ACC and 50 µM AEX. All treatments contained 0.05% DMSO. Analyses were performed by GC-MS/MS on 4-day-old etiolated seedlings. Error bars indicate SD. IAA, oxIAA and oxIAA-GE content in cotyledons (including SAMs) and hypocotyls are presented separately in Supplementary Fig. S8.

Next, we investigated whether AEX affects auxin metabolism and transport *in planta*. Since auxin conjugation and degradation are also essential for the establishment and maintenance of cellular auxin gradients, auxin content and the primary IAA catabolites and conjugates, such as 2-oxindole-3-acetic acid (oxIAA), oxIAA-glucose ester (GE), IAA-aspartate (Asp), IAA-glutamate (Glu) and IAA-GE (Ostin *et al.*, 1998; Kowalczyk and Sandberg, 2001; Kai *et al.*, 2007; Hošek *et al.*, 2012) were measured in cotyledons together with shoot apical meristems (SAMs) and in hypocotyls of 4-day-old dark-grown seedlings. In control seedlings, a substantial amount of free IAA produced in meristems was possibly transported to the hypocotyls, where it was predominantly conjugated into IAA-Glu or metabolized to oxIAA, and subsequently to its non-active stable derivative, oxIAA-GE (Fig. 8 and Supplementary Fig. S8). Upon 50 µM AEX treatment, the amount of free IAA in meristems was maintained, but dropped in hypocotyls to about 30% compared with the control (see Supplementary Fig. S8A, B); in addition, a strong reduction of the IAA conjugates IAA-Asp and IAA-GE was seen, particularly in hypocotyls (Supplementary Fig. S8C). Moreover, while the total amount of oxIAA-GE did not differ from that of control seedlings, the largest fraction accumulated in meristems (85%), and only a small portion in hypocotyls (15%) (Fig. 8 and Supplementary Fig. S8). Since auxin catabolites are not transported across the plasma membrane (Pencik *et al.*, 2013), it is suggested that AEX limits the movement of free IAA produced in meristems, resulting in an apical accumulation of IAA, subsequently oxidized into oxIAA, and converted to oxIAA-GE. Interestingly, the effect of ACC on the spatial distribution of IAA and its catabolites and conjugates was reminiscent of that seen upon AEX treatment (Fig. 8 and Supplementary Fig. S8).

### Changes in global gene expression upon short-term AEX treatment

**Fig. 9.**
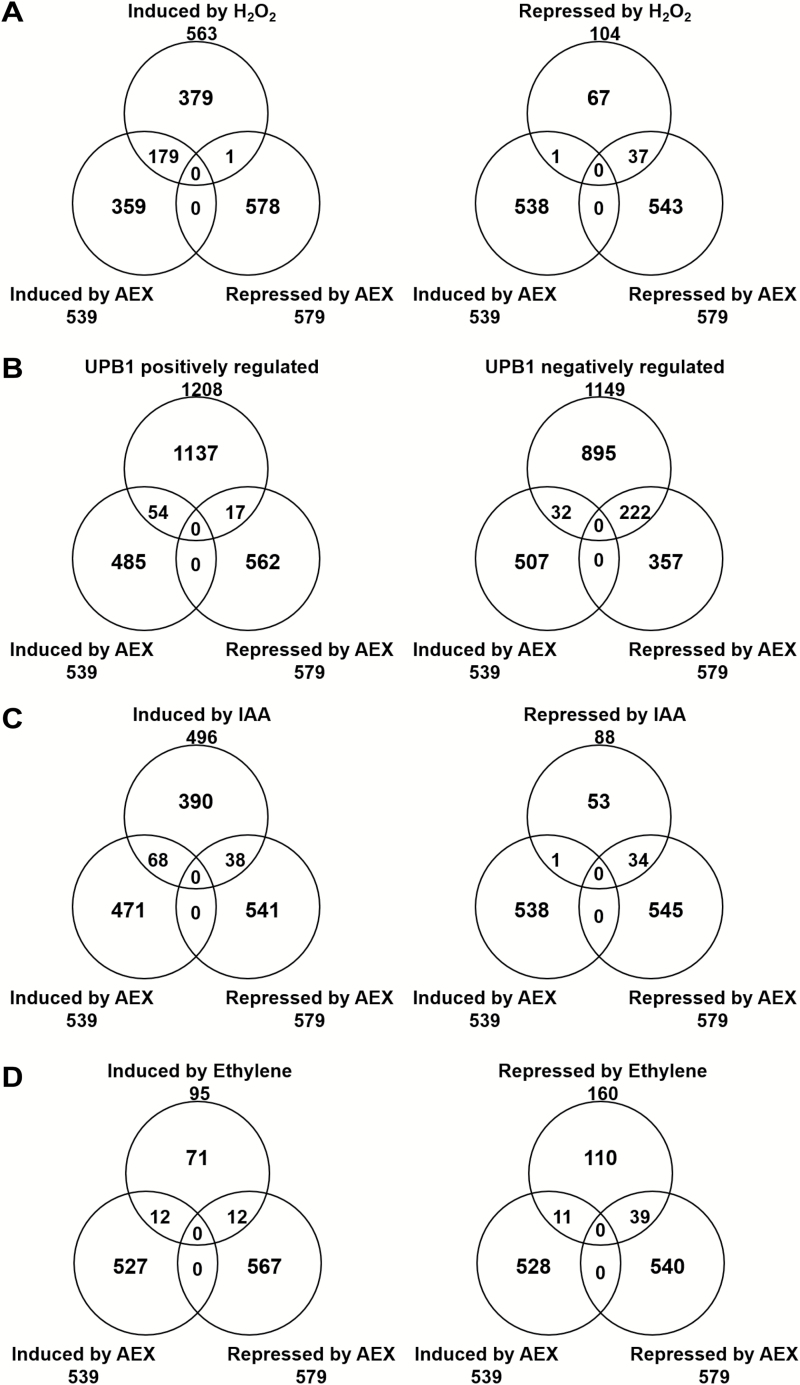
Changes in global gene expression upon short-term AEX treatment. Venn diagrams showing the overlap between transcript dynamics upon AEX treatment (from microarray analysis performed on three independent biological repeats) and published microarray data. Details in Supplementary Tables S2–S5. (A) Induced/suppressed by AEX and H_2_O_2_; the transcriptional profile (>2-fold) of 5-day-old light-grown Col-0 plants treated with H_2_O_2_ (20 mM, 1 h) was from Davletova *et al.* (2005). (B) Induced/suppressed by AEX and UPBEAT1 (UPB1) transcription factor; transcriptional profile of UPB1 regulated genes was from Tsukagoshi *et al.* (2010). (C) Induced/suppressed by AEX and IAA; transcriptional profiles of IAA regulated genes were from Zhao *et al.* (2003), Okushima *et al.* (2005*a*), and Nemhauser *et al.* (2006). (D) Induced/suppressed by AEX and ethylene; transcriptional profiles of ethylene regulated genes were from Alonso *et al.* (2003) and Olmedo *et al.* (2006).

To assess direct effects of AEX, a genome-wide transcript analysis after short-term AEX treatment was performed. RNA was extracted from entire Col-0 seedlings grown for 2.5 d in darkness, and treated for 6 h with 100 µM AEX compared with an untreated control. We identified 539 and 579 genes as up- or down-regulated by AEX compared with the control (see Supplementary Table S2). The Biological Networks Gene Ontology tool (BiNGO) analysis (Maere *et al.*, 2005) showed that genes responding to stimuli and metabolic processes were significantly enriched (see Supplementary Table S3). Comparison of the data with publicly available datasets revealed a link with ROS. Notably, four out of five previously identified hallmarks for the general oxidative stress response (AT1G19020, AT1G05340, AT2G21640, and AT1G57630) (Gadjev *et al.*, 2006) were represented in the AEX-induced set of transcripts. Furthermore, 32% of genes differentially regulated by H_2_O_2_ are shared with AEX, suggesting a strong overlap in response (Fig. 9A and Supplementary Table S4A). Large transcript overlaps were also found when comparing AEX down-regulated genes with genes down-regulated by the bHLH transcription factor UPBEAT1 (UPB1) (Tsukagoshi *et al.*, 2010). More than 38% of genes down-regulated by AEX were shared with those down-regulated by UPB1, while 10% shared up-regulated genes were found (Fig. 9B and Supplementary Table S4B). UPB1 is a transcription factor that negatively regulates root meristem size by repression of a set of class III peroxidases that modulate the balance of ROS at the boundary between the meristematic and elongation zone. In Arabidopsis, there are 73 Class III peroxidase genes (Tognolli *et al.*, 2002), 25 of which were down-regulated by AEX; the majority (70%) overlapped with UPB1 down-regulated peroxidases. Moreover, class III peroxidases are known to modify cell wall structure resulting in cell elongation, through consumption or release of ROS (Passardi *et al.*, 2004). Many Class III peroxidases appear in the top 135 of AEX down-regulated genes with a minimal change of 4-fold along with other cell wall related genes and genes encoding cell wall remodeling enzymes (Supplementary Table S5), some of which have clear effects on cell elongation in a tissue-specific manner (LEUCINE-RICH REPEAT/EXTENSIN1 (LRX1), EXPA7, EXPA18). In order to characterize how AEX may affect auxin response, publicly available microarray data from auxin experiments (Zhao *et al.*, 2003; Okushima *et al.*, 2005*b*; Nemhauser *et al.*, 2006) were analysed. Nearly 25% of auxin-responsive genes were also regulated by AEX, with the majority (102 genes) regulated in the same sense (increased or decreased expression), whereas 39 genes showed an inverse regulatory pattern (Fig. 9C and Supplementary Table S4C). Notably, early auxin-responsive gene families of *Aux*/*IAA*, GretchenHagen-3 (*GH3*), and small auxin-up RNA (*SAUR*) (Abel and Theologis, 1996; Hagen and Guilfoyle, 2002) appeared down-regulated by AEX. Comparison between AEX and transcriptional profiles of ethylene datasets (Alonso *et al.*, 2003; Olmedo *et al.*, 2006) illustrates that the overlap in genes repressed by ethylene and AEX (24%) is larger than the overlap in induced ones (13%); in addition, only 51 genes were regulated in the same sense by AEX and ethylene, indicating that the overlap with ethylene is less than with the signals mentioned above (Fig. 9D and Supplementary Table S4D). Overall, the microarray data indicate a global redox imbalance leading to a ROS induction signature as a prime effect of AEX. The significant overlap of transcripts induced/suppressed by AEX and H_2_O_2_, as well as between AEX and UPB1, suggests that AEX altered ROS homeostasis.

### Induction of reactive oxygen species by AEX

**Fig. 10.**
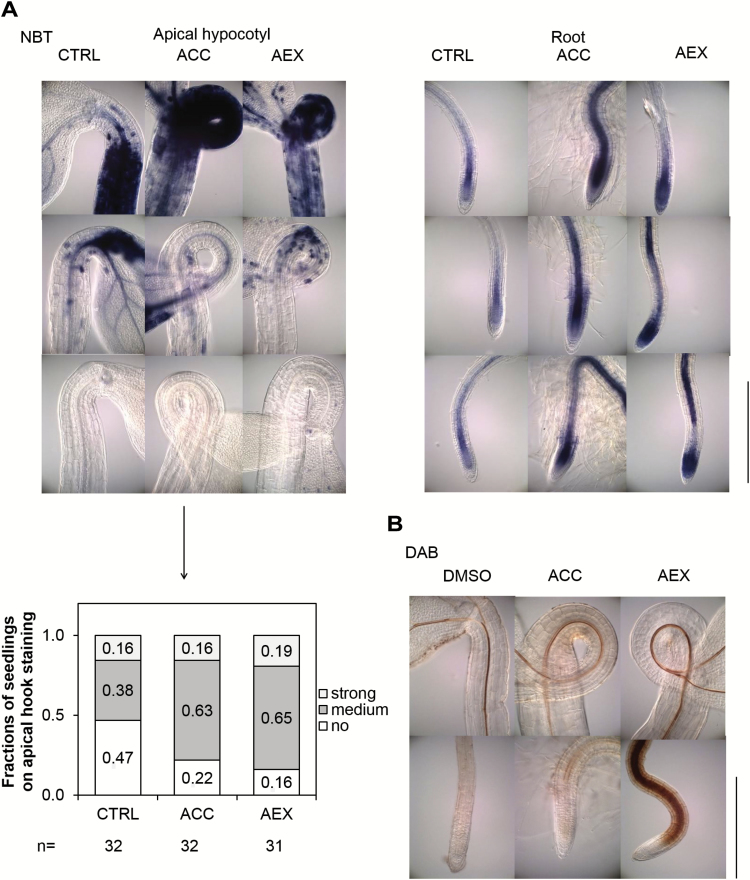
ROS levels are induced by AEX treatment. Col-0 seedlings were continuously grown on medium containing 50 µM AEX for 4 d in darkness compared with untreated (0.05% DMSO) and ACC (10 µM ACC, 0.05% DMSO)-treated seedlings. (A) Images of 4-day-old etiolated seedlings stained for O_2_^−^ using nitroblue tetrazolium (NBT) and summary of frequencies of staining intensity. The degree of staining in the apical part of the hypocotyl was classified as strong, medium, or no staining. Fractions of seedling staining on the apical hook are shown with sample sizes indicated under the graph. (B) Images of 4-day-old etiolated seedlings stained for H_2_O_2_ using diaminobenzidine (DAB). Scale bar: 0.5 mm.

In order to obtain direct proof that the ROS balance was altered by AEX, both nitroblue tetrazolium (NBT) and diamino benzidine (DAB) staining was performed on 4-day-old seedlings, reflecting endogenous levels of superoxide (O_2_^−^) and H_2_O_2_, respectively. NBT staining was mainly detected in the apical regions of hypocotyl and root (Fig. 10A). The fraction of seedlings stained in the apical part of the hypocotyl was significantly larger in AEX-treated seedlings as compared with the control (AEX: sum of strong and medium=0.84; control: 0.54) (Fig. 10B). ACC treatment resulted in staining patterns comparable to AEX (0.79). Furthermore, both ACC and AEX induced the O_2_^−^ level in the root, particularly in the root tip and the vasculature (Fig. 10A). In contrast, the DAB staining did not result in significant differences in the apical region of hypocotyls, while staining was significantly increased in roots treated with AEX compared with both untreated and ACC-treated seedlings (Fig. 10B). In accordance with the microarray data, these results demonstrate that the ROS level is enhanced by AEX. Moreover, DAB staining of AEX-treated seedlings was stronger in the elongation zone than in the meristem, while being significantly weaker in the epidermis of the elongation zone as compared with inner cell types.

### Herbicidal properties of AEX

**Fig. 11.**
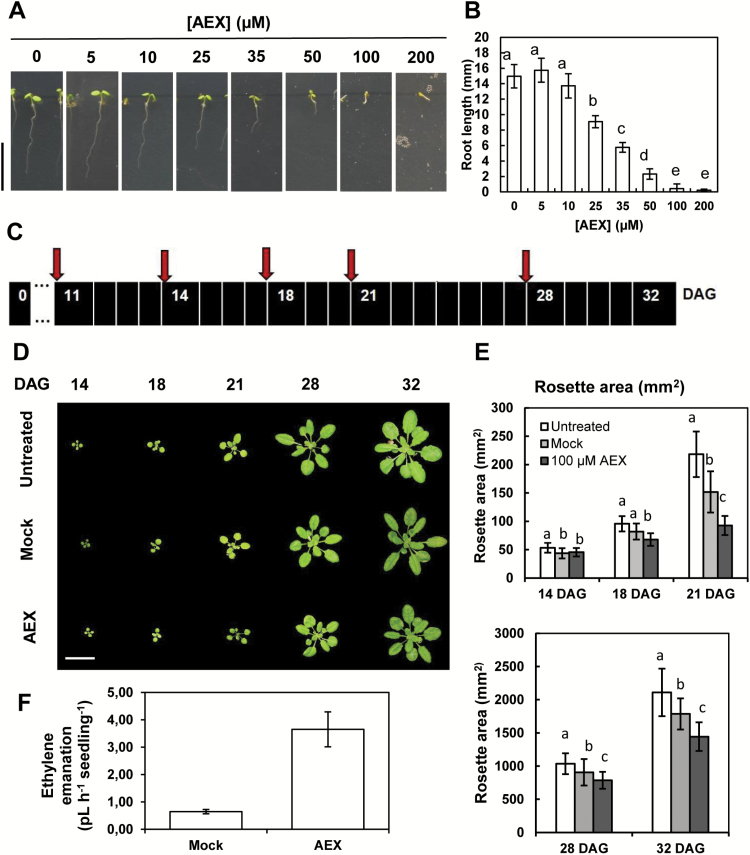
AEX exhibits herbicidal properties. (A) Representative phenotypes of 7-day-old light-grown seedlings supplemented continuously with increasing concentrations of AEX (10<*n*<40; scale: 10 mm). (B) Average root lengths of 7-day-old light-grown seedlings presented in (A). Bars represent means±SD. Different letters represent means that are significantly different based on the Kruskal–Wallis test (*P*<0.05) followed by *post hoc* Wilcoxon’s rank sum analyses (*P*<0.05). (C) Scheme of frequency of spray treatments applied for experiments in (D, E). Red arrows represent timing of spraying. After spraying a photograph was taken to visually assess and quantify phenotypic effects. (D) Representative pictures of Arabidopsis rosette phenotypes after spraying with 0.1–0.2% Tween 20 (Mock), 0.1–0.2% Tween 20 + 100 µM AEX (AEX) or no spraying (Untreated) (Scale: 20 mm). (E) Averages of rosette area at various growth stages. Bars represent means±SD. For each time point, statistical analysis was done separately, where different letters represent means that are significantly different based on the Kruskal–Wallis test (*P*<0.05) followed by *post hoc* Wilcoxon’s rank sum analyses (*P*<0.05). (F) Ethylene emanation of 7-day-old light-grown seedlings continuously treated with 50 µM AEX. Means±SD are shown.

Given the growth inhibitory effects on seedling development and the enhancement of ROS levels by AEX in darkness, possibly through the modulation of auxin homeostasis, AEX might act as an auxin-like herbicide (Grossmann, 2010). Moreover, its quinoline backbone shares a strong structural similarity with the quinoline carboxylate auxin-like herbicides quinmerac (7-chloro-3-methylquinoline-8-carboxylic acid) and quinclorac (3,7-dichloroquinoline-8-carboxylic acid) (Grossmann and Kwiatkowski, 1995; Grossmann and Scheltrup, 1998). Moreover, quinoline carboxamide and carboxylate AEX analogs were able to induce similar phenotypic effects and enhance shoot gravitropism in etiolated seedlings, indicating that the quinoline core could be the effective principle (see Supplementary Table S6 and Supplementary Fig. S9). A dose–response assay was carried out on 7-day-old light-grown seedlings to assess the growth inhibitory effects of AEX. Thirty-five micromolar AEX effectively inhibited primary root growth by 60%, while concentrations greater than 100 µM resulted in severe developmental effects, eventually leading to seedling death (Fig. 11A, B). To determine whether AEX could be employed as a post-emergence herbicide, soil-grown Arabidopsis seedlings were subjected to foliar spraying (Fig. 11C–E). To mimic spray formulations used for quinclorac (and/or quinmerac), a non-ionic surfactant was added to a solution of 100 µM AEX, to enhance foliar uptake (Tween 20; 0.1–0.2% v/v) (Woznica *et al.*, 2003; Van Eerd *et al.*, 2005; Lovelace *et al.*, 2007; Hoagland *et al.*, 2011). Higher concentrations impeded AEX solubility, while higher levels of Tween 20 affected plant growth. AEX treatment significantly reduced rosette area compared with untreated plantlets (Fig. 11D, E). This inhibitory effect on rosette growth, however, was smaller compared with continuously treated plants, which is probably related to reduced foliar uptake as compared with root penetration (Fig. 11A, B and Supplementary Fig. S10). Interestingly, at 50 µM a concomitant 5-fold increase in ethylene levels was observed, indicating a stimulation of ethylene biosynthesis in light-grown seedlings (Fig. 11F).

## Discussion

### ACCERBATIN, a quinoline carboxamide that exacerbates ethylene effects in etiolated seedlings, acts in parallel to the ethylene pathway

We recently screened the DIVERSet™ library from ChemBridge™ (http://www.chembridge.com/index.php, last accessed 16 July 2017), which contains 12 000 chemicals with broad structural diversity, for compounds altering the ACC-induced triple response phenotype of etiolated Arabidopsis seedlings (Hu *et al.*, 2014). A number of compounds, including a quinoline carboxamide compound called accerbatin for its *A*CC *ex*acerbating effect (AEX; Fig. 1A), were chosen for further investigation. Here, we present a detailed study of the effects of AEX at the cellular and whole plant level, and propose a mechanism of action, based on a number of chemical, genetic, molecular and physiological analyses.

Since application through the medium resulted in a conspicuous shoot phenotype, AEX or a metabolite thereof appeared to be effectively taken up and transported throughout the plant body. An LC-MS-based global metabolomics study in etiolated seedlings and an NMR analysis of the compound *in vitro* indicated that AEX probably remains stable *in planta* (Supplementary Protocols S1 and S2). The only cleavage compound found (the prevalence of which could not be assessed precisely, but which was assumed to be very low, as also suggested by NMR analysis) resulted from loss of the C_4_H_2_NF_2_ fragment of AEX through hydrolysis of the amide bond, essentially leaving the quinoline core with aromatic substitution on C3 (Fig. 1). In addition, phenotypic analysis along with assessment of effects on shoot gravitropism of quinoline carboxamide and carboxylate AEX analogs suggested that the quinoline core could be the effective principle (see Supplementary Table S6 and Supplementary Fig. S9). However, this was not supported by analog G, which was inactive. Therefore, it can be concluded that AEX is largely stable and acts as such *in planta*.

Given that AEX issued from a screen for altered triple response characteristics, initial experiments were directed towards its possible role in ethylene biosynthesis or signaling. AEX triggered triple response-like characteristics or exacerbated the triple response in ethylene biosynthesis and signaling mutants (Fig. 5B), reminiscent of the phenotype of WT seedlings treated with AEX or the combination of AEX and ACC (Fig. 1B). Furthermore, AEX triggered apical hook development in *hls1-1*, suggesting AEX action downstream of HLS1 (see Supplementary Fig. S4A, C). The partial hook was similar to the restored hook curvature in the *HLS1 suppressor* mutant (*hss1)/arf2*, with the auxin responsive transcription factor *ARF2* acting downstream of *HLS1*, a putative *N*-acetyltransferase (Li *et al.*, 2004). When combining ACC with AEX, the dynamics of *hls1-1* hook development was very similar to that observed in AEX-treated seedlings (Supplementary Fig. S4C). Hence, it was concluded that AEX acts downstream or independent of ethylene signaling. Ethylene independence was further suggested by analysis of the *EBS::GUS* reporter, in which AEX did not substantially affect expression (Supplementary Fig. S3B).

Analysis of cellular effects of AEX indicated similarities and differences of ethylene and AEX targets. AEX inhibited elongation, but not lateral expansion of the hypocotyl as ethylene does (Fig. 3A–D). Furthermore, AEX strongly inhibited root meristematic activity (see Supplementary Fig. S3A), resulting in a short meristem (Fig. 4A–D), as well as inhibited cell size in the elongation zone, while ethylene had a limited effect on the meristem, but affected cell elongation similarly to AEX (Fig. 4E, F). Moreover, AEX resulted in suppressed ACC/ethylene-induced lateral expansion (Fig. 3C, D). In addition, AEX inhibited root hair emergence and outgrowth as opposed to the induction typically seen for ethylene (Fig. 4G, H). Altogether, these results support the contention that AEX acts in parallel to the ethylene pathway rather than downstream of it.

### AEX interferes with auxin metabolism at different levels

Analysis of *pCYCB1;1::DB-GUS* expression revealed an opposite effect of AEX on the root apex *versus* the apical hook (see Supplementary Fig. S3A), suggesting that AEX differentially controls cell cycle activity, probably depending on the impact on IAA homeostasis. A similar case of spatial differences in cell cycle control was found in heavy metal exposed roots, in which meristematic activity in the main root was inhibited, but the cell division activity leading to new lateral roots was induced (Pasternak *et al.*, 2005*a*,b). These changes in root patterning suggested an auxin redistribution. Likewise, AEX is proposed to have an impact on auxin homeostasis, primarily acting at the shoot, and affecting the root as a consequence thereof.

The concentration of auxin within a plant cell is regulated both by the rate of its metabolism (synthesis, conjugation, catabolism) and the capacity and rate of its transport, together regulating cellular auxin homeostasis. Essentially the above-mentioned observations, together with the fact that AEX acts downstream of HLS1, suggest either an enhanced auxin catabolism or an interference with auxin efflux transport. The latter was supported by a dose-dependent accumulation of [^3^H]NAA in tobacco BY-2 cells, indicating that AEX can block auxin export (Fig. 7). Furthermore, by analysing IAA, IAA conjugates, and the major IAA catabolites in AEX-treated etiolated seedlings, we hypothesized that AEX limited movement of free IAA produced in meristems since its final catabolite, oxIAA-GE, largely accumulated there (Fig. 8 and Supplementary Fig. S8). The elevated production of oxIAA-GE suggested enhanced oxidative activity triggered by AEX. Since auxin catabolites are not transported across the plasma membrane (Pencik *et al.*, 2013), the very high amount of ox-IAA-GE indicated that only a small portion of IAA is transported to the hypocotyl. The altered auxin metabolism in the shoot could consequently lead to disrupted auxin homeostasis in the root, because of the minimized basipetal transport of active auxins. Recently, ROS were shown to induce the oxidation of IAA to oxIAA, in order to remove high levels of active auxin from the root apex to attenuate auxin signaling and maintain auxin homeostasis (Peer *et al.*, 2013; Pencik *et al.*, 2013). The link between AEX and ROS was largely supported by our microarray data and NBT/DAB stains. From the microarray analysis, indirect support was offered by more than one-third overlap with H_2_O_2_-induced transcripts in the AEX up-regulated gene set (Fig. 9A). Direct evidence for induction of ROS by AEX came from the NBT/DAB stains, where an enhanced accumulation of O_2_^−^ was observed in the apical regions of hypocotyl and root and an enhanced accumulation of H_2_O_2_ above the meristem towards the root differentiation zone (Fig. 10). Studies have shown that auxin-type herbicides might act through induction of H_2_O_2_ (Grossmann *et al.*, 2001; Peer *et al.*, 2013). Greatly increased ROS accumulation induced by AEX could disrupt the redox homeostasis, further oxidize IAA, and hence lower the IAA level, and ultimately diminish the meristematic cell activity as reported in the tomato (*Solanum lycopersicum*) *diageotropica* (*dgt*) mutant (Ivanchenko *et al.*, 2013). It was proposed that once the ratio of H_2_O_2_ to O_2_^−^ reaches its maximal level, cell proliferation ceases, and cells differentiate (Tsukagoshi *et al.*, 2010). The reduced meristem size might result from reduction in cell wall extensibility of developing root cells, resulting from ROS accumulation (Büntemeyer *et al.*, 1998). In the microarray dataset, a group of cell wall proteins whose activity directly enhances cell wall extensibility, such as PROLINE RICH PROTEIN 3 (PRP3), LEUCINE-RICH REPEAT/EXTENSIN 1 (LRX1) and expansins (Cosgrove, 2005), were down-regulated (see Supplementary Table S5). Particularly interesting is that in the AEX down-regulated gene set, more than one-third of the genes overlapped with root-specific UPBEAT1 (UPB1) down-regulated transcripts, including a large set of peroxidases (Tsukagoshi *et al.*, 2010) (Fig. 9B). In addition, ectopic UPB1 expression conferred shortening of root meristem and overall length as well as significant decrease in cortex cell number, phenotypes mimicked by AEX. Ectopic UPB1 expression also resulted in enhanced H_2_O_2_ accumulation above the root meristem accompanied by a decreased O_2_^−^ in the meristem to maintain ROS homeostasis, as seen for AEX. Therefore, UPB1 is a candidate target of AEX.

Based on the above-mentioned findings we propose a model in which AEX interferes with auxin transport from its major biosynthesis sites, the SAM and cotyledons. This is either the direct consequence of poor basipetal IAA transport from the meristematic region, or indirectly linked to excessive IAA oxidation. The auxin transporters affected by AEX could be PIN-FORMED (PIN)1 and ATP-BINDING CASSETTE B/P-GLYCOPROTEIN/MULTIDRUG RESISTANCE (ABCB/PGP/MDR)19, primary mediators of shoot basipetal polar auxin transport (PAT) (Gälweiler *et al.*, 1998; Noh *et al.*, 2001). Given the central role of PAT, with the major auxin flux directed from shoot to root, a distortion of auxin homeostasis in the shoot is expected to have severe consequences in the root. This was reflected by enhanced ROS staining in the root tip, probably related to an imbalance in auxin (Fig. 10). Microarray data supported accumulation of ROS in AEX-treated seedlings (Fig. 9A, B). On the other hand, excessive IAA oxidation by NADPH oxidases such as RbohD enhances ROS generation and results in increased levels of oxIAA, which is not transported from cell to cell (Peer *et al.*, 2013). In the root tip, auxin accumulation results from PAT from the shoot and auxin synthesis at the root meristem (Ljung *et al.*, 2005). The strongly reduced stelar auxin flux toward the root tip probably results in a local increase in auxin synthesis and subsequent ROS accumulation, known to limit the size of the root meristem (Tsukagoshi *et al.*, 2010; Ivanchenko *et al.*, 2013), as seen upon AEX treatment (Fig. 4C, D). Both basipetal transport and lateral distribution of auxin, mediated by the auxin transport facilitators PIN2, PIN3, and PIN7, are critical for controlling cell division and root meristem size (Blilou *et al.*, 2005). PIN(s) could be the candidate auxin transporters affected by AEX; however, effects of AEX on ABCB(s) transporters or their interactions with PIN(s) cannot be excluded (Blakeslee *et al.*, 2007; Mravec *et al.*, 2008). The inhibitory effects on root hair initiation and growth triggered by AEX (Fig. 4G, H) could result from a transiently suppressed auxin signal caused by increased ROS production (Blomster *et al.*, 2011). A recent study also suggests that the impaired root hair growth in multiple *pin* loss-of-function mutants most likely results from the imbalance in auxin homeostasis (Rigas *et al.*, 2013).

Altered auxin accumulation was also reflected in altered gravitropism triggered upon AEX treatment and may be related to an alteration in endomembrane trafficking, affecting auxin transport. A successful example is the identification of gravacin as a gravitropism and vacuolar transport inhibitor from a chemical genetics screen, which linked the altered gravity response phenotype with vesicular trafficking. ABCB19 was identified as a target for gravacin (Rojas-Pierce *et al.*, 2007; Surpin *et al.*, 2005). The link of AEX-triggered hypocotyl gravitropic response with endomembrane trafficking could be tested with tonoplast-specific markers, such as GFP:γ-TIP and GFP:δ-TIP (Cutler *et al.*, 2000).

### AEX exhibits biological properties reminiscent of auxin-like herbicides

Quinoline derivatives have very different biological properties in several kingdoms, including antibacterial (Shivaraj *et al.*, 2013), antimalarial (Raynes *et al.*, 1996; Narayan Acharya *et al.*, 2008), antitumor (Isaacs *et al.*, 2006), and herbicidal (Grossmann and Kwiatkowski, 1995; Grossmann and Scheltrup, 1998) action. Similar to other auxin herbicides and IAA, at supra-optimal concentrations in dicot plants, quinoline carboxylates stimulate ethylene production in the light via the induction of *ACS* (Grossmann and Kwiatkowski, 1995; Grossmann and Scheltrup, 1998), ultimately leading to leaf epinasty, tissue swelling and senescence (Grossmann, 2003). Subsequently, ABA levels rise, resulting in stomatal closure, which leads to decreased respiration and accumulation of ROS (Grossmann *et al.*, 2001). Likewise, AEX-treated light-grown plants displayed severe growth inhibition, accelerated senescence and vitrification along with a strong induction of ethylene biosynthesis (Fig. 11A–F and Supplementary Fig. S10). Though significant, the reduced uptake upon foliar spraying and lower dosage compared with current available products containing quinmerac/quinclorac resulted in a partial growth inhibition. It should also be noted that quinmerac is used in combination with other herbicides such as chloridazon (Fiesta®, BASF, Belgium) and metazachlor (Butisan Top®, BASF, Belgium), boosting the effect of the formulation (Böger *et al.*, 2000; Lutman *et al.*, 2008). Additional adjuvants and surfactants could further enhance uptake of AEX and increase solubility, allowing higher doses (Woznica *et al.*, 2003; Van Eerd *et al.*, 2005). Furthermore, AEX affected auxin signaling (see Supplementary Fig. S3C) and metabolism (Fig. 8 and Supplementary Fig. S8), inhibited auxin efflux (Fig. 7), and induced ROS accumulation in etiolated seedlings (Fig. 10), providing additional parallels to auxin-like herbicides.

The observed traits demonstrate that AEX might act on the molecular effector(s) of the quinoline carboxylate-type auxins. Quinmerac, quinclorac, and other auxin herbicides were shown to target the TIR1/AFB family of auxin receptors, as is the case for IAA (Dharmasiri *et al.*, 2005; Calderón Villalobos *et al.*, 2012; Lee *et al.*, 2014; Prigge *et al.*, 2016). A docking position for AEX in the binding pocket of TIR1 was predicted *in silico* (see Supplementary Fig. S11), but the orientation in the binding cavity differed from IAA and other synthetic auxins, indicating that the putative docking position might be a false positive. Moreover, AEX does not contain the required carboxyl group shared with IAA and other synthetic herbicides, which is involved in binding (Grossmann, 2010; Calderón Villalobos *et al.*, 2012). It cannot be ruled out that AEX is enzymatically converted *in planta*, although our LC-MS global metabolomics study did not support such conversion (Supplementary Protocol S1). Interestingly, it was shown that picolinate and quinoline carboxylate-type auxins preferentially bind AFB4 and AFB5, probably due to differences in the binding pocket (Calderón Villalobos *et al.*, 2012; Lee *et al.*, 2014; Prigge *et al.*, 2016). Thus, AEX could preferentially bind these divergent AFB proteins.

Further investigation of AEX can help to resolve issues linking ROS and auxin homeostasis in plant development. In order to gain insight into the auxin transporters that are affected by AEX, inhibitory effects of AEX on auxin transport mediated by recombinant PIN(s) and ABCB(s) expressed in *Schizosaccharomyces pomb*e could be screened for (Yang and Murphy, 2009). Additional work on the herbicidal action will aid in the discovery of molecular target(s) of AEX and might provide tools useful in the agricultural and horticultural industry. Current work is focusing on the identification of AEX targets using a forward genetics screen to identify mutants with reduced or enhanced sensitivity to AEX.

## Supplementary data

Supplementary data are available at *JXB* online.

Fig. S1. Sample preparation for determination of IAA, its conjugates and catabolites.

Fig. S2. NMR DATA.

Fig. S3. Histochemical staining in four-day etiolated seedlings of GUS-reporter lines.

Fig. S4. AEX-regulated apical hook development of etiolated seedlings grown on vertical plates

Fig. S5. Phenotypic effects of AEX on auxin mutants and of AEX in combination with auxins and auxin transport inhibitors on the wild-type.

Fig. S6. Response of Col-0, *msg2-1* and *nph4-1arf19-1* after growth and reorientation on vertically standing plates. 

Fig. S7. [^3^H]NAA accumulation kinetics in tobacco BY-2 cells upon 100 µM AEX or/and 100 µM ACC treatments.

Fig. S8. GC-MS/MS determination of the endogenous content of IAA, IAA catabolites and IAA conjugates of four-day etiolated seedlings treated with AEX.

Fig. S9. Effects of AEX and its analogs on phenotypes of etiolated seedlings and on the gravitropic response of hypocotyls.

Fig. S10. Phenotype of light-grown plants.

Fig. S11. *In silico* docking simulation of IAA and AEX.

Table S1. Summary of statistical analysis.

Table S2. Genes regulated by 6 hours’ AEX treatment.

Table S3. Gene Ontology of AEX-regulated genes.

Table S4. Common genes regulated by AEX and other arrays.

Table S5. Cell wall related genes which expression decreased by minimal 4-fold after 6 hours of AEX treatment.

Table S6. The minimal tested concentration (10 or 50 µM) of AEX analogs to induce the apical hook curvature.

Protocol S1. ACCERBATIN stability *in vivo* determined by LC-MS profiling.

Protocol S2. AEX stability *in vitro* determined by NMR.

Protocol S3. Kinematic and genetic analysis of the effect of AEX on hook development.

## Author contributions

DVDS, FV and YH designed experiments; YH, TD (experiments in Figs 3A–E, 4A–H, and 11A–F, and Supplementary Figs S3A, S10, and S11), and DS (experiments in Fig. S4) performed the phenotypic, physiological and genetic characterizations; KH and JP performed the analysis of auxin and auxin metabolites; PK and JP were responsible for auxin accumulation experiments on BY-2 cell-suspension culture; AC assisted with interpretation of the oxidative stress response; SC provided essential research materials; DB and JM performed the NMR analysis; KM and WB performed metabolite profiling; and TD performed the statistical analysis. Research coordination was done by DVDS. DVDS, YH, and TD wrote the manuscript, and all authors commented on the manuscript.

## Supplementary Material

Supplementary_Tables_S1_S5

Supplementary_Figures_S1_S11_Table_S6_Protocols
