# Supplementary material for: ACCERBATIN, a small molecule at the intersection of auxin and reactive oxygen species homeostasis with herbicidal properties
Source: J Exp Bot. 2017 Jul 26;68(15):4185–203. doi: 10.1093/jxb/erx242 (PMC5853866; doi:10.1093/jxb/erx242)
Supplement: Supplementary_Figures_S1_S11_Table_S6_Protocols [file erx242_suppl_supplementary_figures_s1_s11_table_s6_protocols.pdf]

Supplementary Fig. S1 Sample Preparation for Determination of IAA, its Conjugates and Catabolites. Illustration of where the cotyledon (with SAM) and hypocotyl were separated. Etiolated seedlings were grown on 1/2 MS medium containing 1% sucrose supplemented with 0.05% DMSO (CTRL), 10 µM ACC and 50 µM AEX for 102-hour. All the treatments contained 0.05% DMSO. Scale bar = 1 cm.

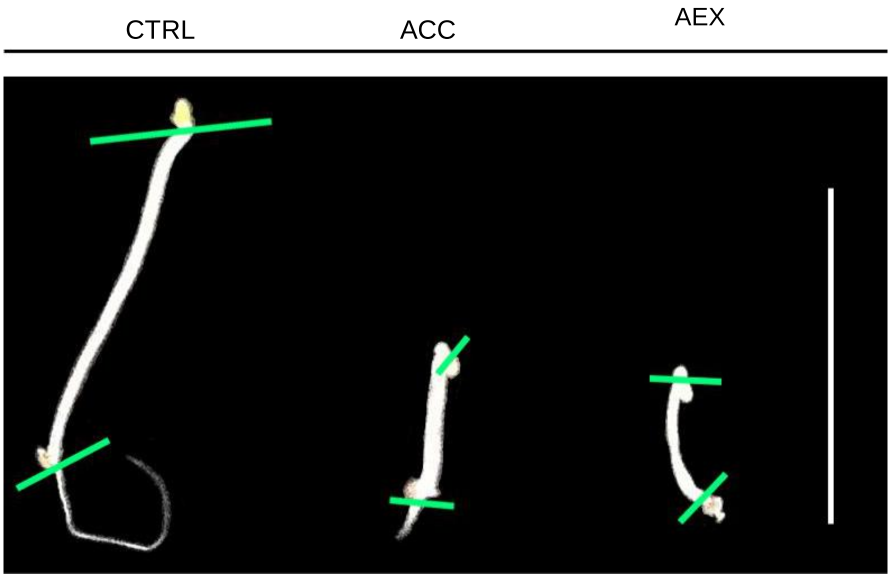

Supplementary Fig. S2 NMR Data (See Also Supplementary Notes S2). **(A)** AEX structural formula and numbering used throughout the NMR assignment procedure. **(B)** Overview of the general 1D1H assignment of AEX (25°C, 500 MHz). **(C)** Overview of the three samples measured at room temperature (25°C, 500 MHz). **(D)** Overview of the AEX temperature study at 50°C after zero, six and 12 hours time (500 MHz). **(E)** Overview of the pH study on AEX (25°C, 500 MHz). (**F)** Overview of the pH study on AEX prior heated at 80°C (25°C, 500 MHz).

A

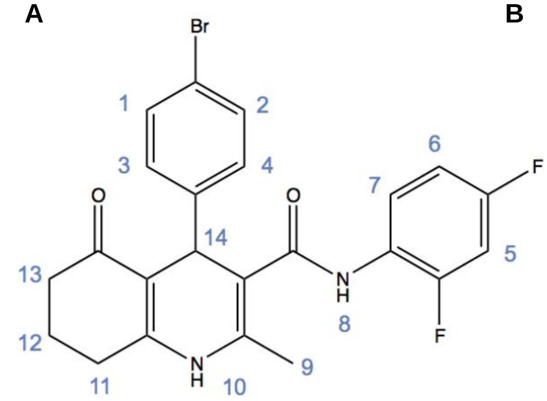

B

δ 1H (ppm)	multiplicity	Integral	# protons	Annotation
1,9/2,0	multiplet	1,13; 0,87	2	12
2,15	singlet	3,26	3	9
2,31	multiplet	2,37	2	11
2,54	multiplet	2,82	2	13
6,88	triplet	0,86	1	5
6,97	triplet of doublets	0,89	1	6
7,2	doublet	2,02	2	3 & 4
7,36	doublet	2	2	1 & 2
7,45	multiplet	0,89	1	7
8,75	broad singlet	0,61	1	8 or 10
9,12	broad singlet	0,64	1	8 or 10
		**Total**	18	

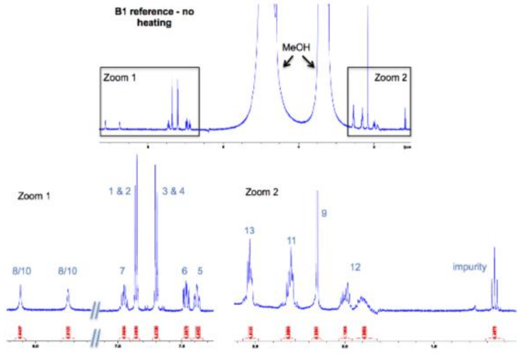

C

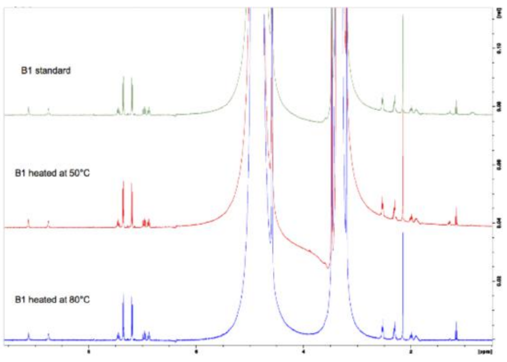

D

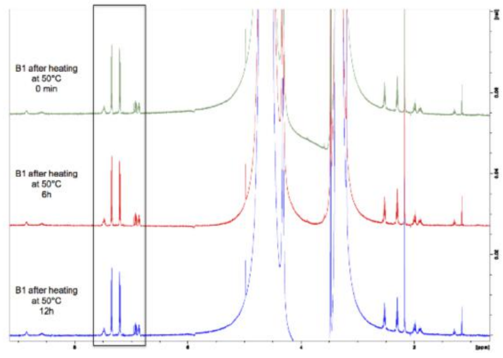

E

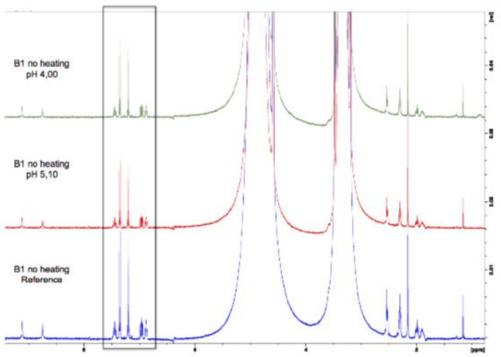

F

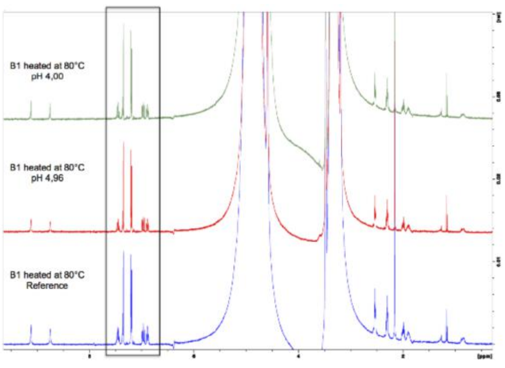

Supplementary Fig. S3 Histochemical Staining in four-day Etiolated Seedlings of GUS-Reporter Lines. GUS Reporter Lines Were Grown On 1/2 MS Medium Containing 1 % Sucrose Supplemented with 0.05% DMSO (CTRL), 10 µM ACC, 50 µM AEX or/and 10 µM ACC. All treatments contained 0.05% DMSO. **(A)** pCYCB1;1::DB::GUS; **(B)** EBS::GUS; **(C)**. DR5::GUS. Scale bar = 100 µm.

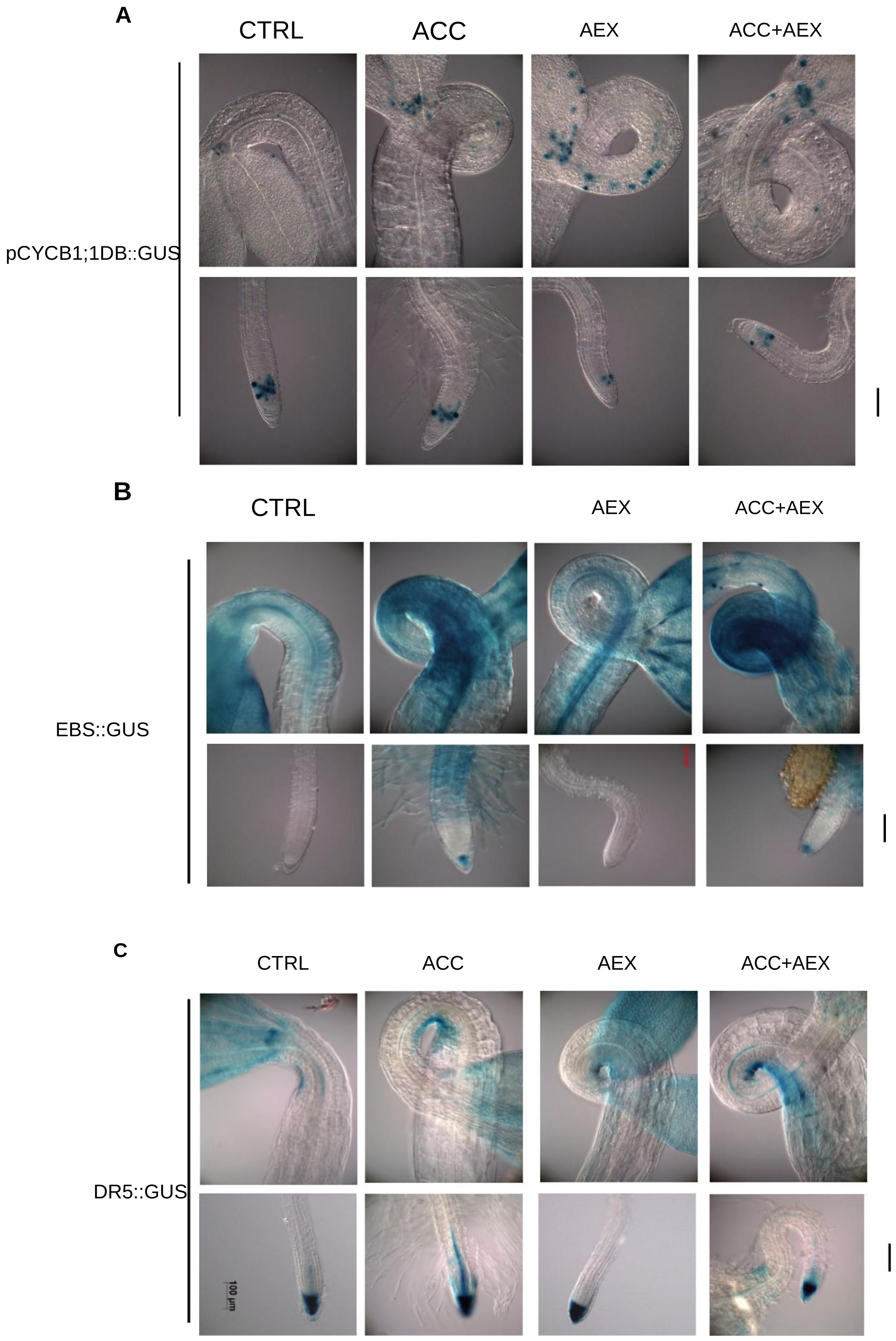
A
CTRL
ACC
AEX
ACC+AEX
pCYCB1;1DB::GUS
B
CTRL
AEX
ACC+AEX
EBS::GUS
C
CTRL
ACC
AEX
ACC+AEX
DR5::GUS

Supplementary Fig. S4 AEX-regulated apical hook development of etiolated seedlings grown on vertical plates. (A) Phenotypic effects of 0.05% DMSO (CTRL), 10 µM ACC, 50 µM AEX or/and 10 µM ACC on the apical hook curvature of etiolated seedlings at 60 hours after germination. The individual photographs were cropped without changing the scale. Scale bar = 5 mm. Kinetics of hook development in Col-0 **(B)** and *hls1-1* **(C)** seedlings grown on 0.05% DMSO (CTRL), 10 µM ACC, 50 µM AEX, 10 µM ACC + 50 µM AEX. Yellow vertical lines represent the transition between developmental phases. The apical hook of wild-type in control medium forms shortly after germination, until bending reaches a plateau c. 170° corresponding to the formation phase (F); the maintenance phase (M) spans a period of 30-60 hours (at day two and day three); subsequently, the apical hook starts opening (opening phase (O)). All treatments contained 0.05% DMSO. Data are presented as mean ± SD. Angle of AEX-treated *hls1-1* was compared to CTRL *hls1-1* at timepoint 24 hours by means of a Wilcoxon rank sum test ($P < 0.005$ (**); 6 > n > 10). Experiments were performed twice, with comparable kinetics.

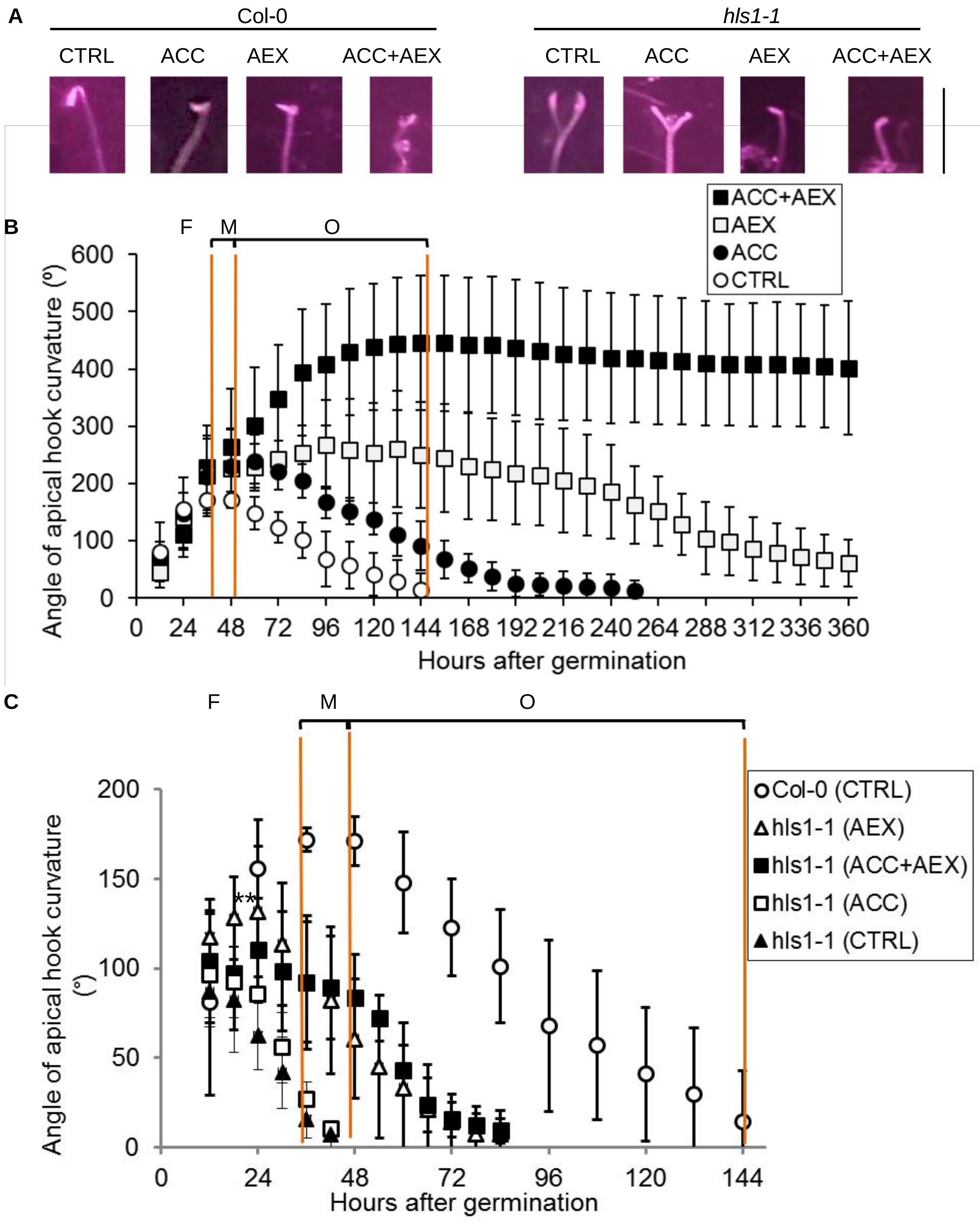

A
Col-0
hls1-1
CTRL
ACC
AEX
ACC+AEX
B
F
M
O
ACC+AEX
AEX
ACC
CTRL
Angle of apical hook curvature (º)
Hours after germination
C
Col-0 (CTRL)
hls1-1 (AEX)
hls1-1 (ACC+AEX)
hls1-1 (ACC)
hls1-1 (CTRL)
Angle of apical hook curvature (°)

Supplementary Fig. S5. **Phenotypic Effects of AEX on Auxin Mutants and of AEX in Combination with Auxins and Auxin Transport Inhibitors on the Wild Type. (**A) Phenotypes of selected auxin mutants in the presence of 50 µM AEX. Auxin signaling mutants: arf2-6, nph4-1arf19-1 and axr3-1; auxin transport mutants: aux(s)lax3, aux1-7, 35S::PIN1, pin3-3, rcn1-1, pid(s), wag(s), pgp4-1 and abcb1abcb19. (B) Phenotypes of Col-0 in the presence of 50 µM AEX and 0.5 µM IAA, 0.1 µM 2,4-D, 10 µM 1-NOA or 10 µM NPA.

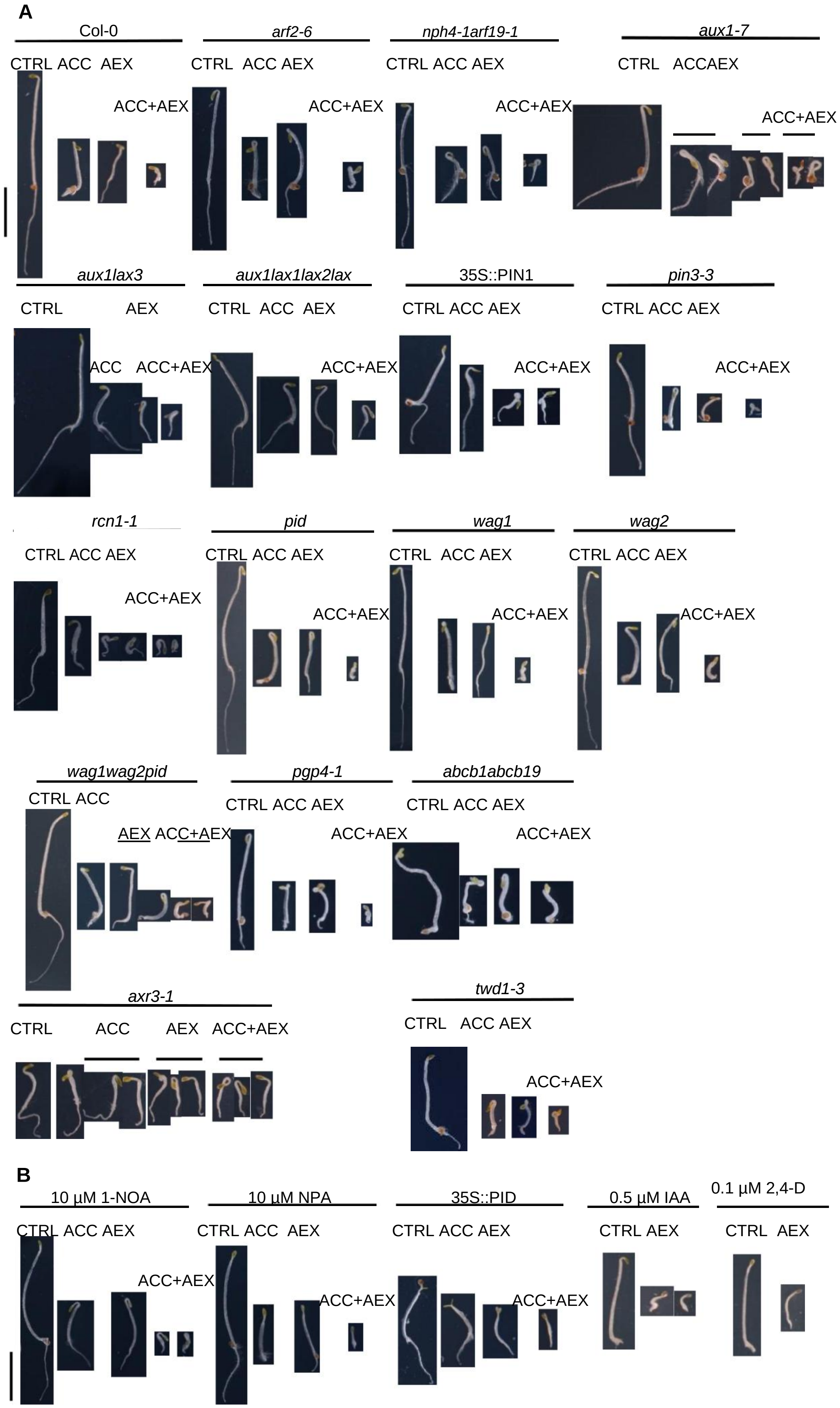
A
Col-0
CTRL ACC AEX
ACC+AEX
arf2-6
CTRL ACC AEX
ACC+AEX
nph4-1arf19-1
CTRL ACC AEX
ACC+AEX
aux1-7
CTRL ACCAEX
ACC+AEX
aux1lax3
CTRL AEX
ACC ACC+AEX
aux1lax1lax2lax
CTRL ACC AEX
ACC+AEX
35S::PIN1
CTRL ACC AEX
ACC+AEX
pin3-3
CTRL ACC AEX
ACC+AEX
rcn1-1
CTRL ACC AEX
ACC+AEX
pid
CTRL ACC AEX
ACC+AEX
wag1
CTRL ACC AEX
ACC+AEX
wag2
CTRL ACC AEX
ACC+AEX
wag1wag2pid
CTRL ACC
AEX ACC+AEX
pgp4-1
CTRL ACC AEX
ACC+AEX
abcb1abcb19
CTRL ACC AEX
ACC+AEX
axr3-1
CTRL ACC AEX ACC+AEX
twd1-3
CTRL ACC AEX
ACC+AEX
B
10 μM 1-NOA
CTRL ACC AEX
ACC+AEX
10 μM NPA
CTRL ACC AEX
ACC+AEX
35S::PID
CTRL ACC AEX
ACC+AEX
0.5 μM IAA
CTRL AEX
0.1 μM 2,4-D
CTRL AEX

Supplementary Fig. S6. Response of Col-0, *msg2-1* and *nph4-1arf19-1* after Growth and Reorientation on Vertically Standing Plates. Seedlings were grown in darkness. On day two after germination, plates were rotated and the average reorientation angle of the hypocotyl was calculated. Data are mean values of at least six seedlings. Seedlings were grown on 1/2 MS medium containing 1% sucrose in the presence of 0.05% DMSO (CTRL), 10 µM ACC, 50 µM AEX or 10 µM NPA. All treatments contained 0.05% DMSO. 90°corresponds with the new direction of the gravity vector. Data are presented as mean ± SD. Angles of AEX-treated *msg2-1* and *nph4-1arf19-1* were compared with their untreated equivalents at four hours and eight hours after reorientation by means of a Wilcoxon rank sum test (for *msg2-1*: $P > 0,05$ (4 hours); $P > 0,05$ (8 hours); for *nph4-1arf19-1*: $P > 0,05$ (4 hours); and $P > 0,05$ (8 hours)). In all conditions: n = 10.

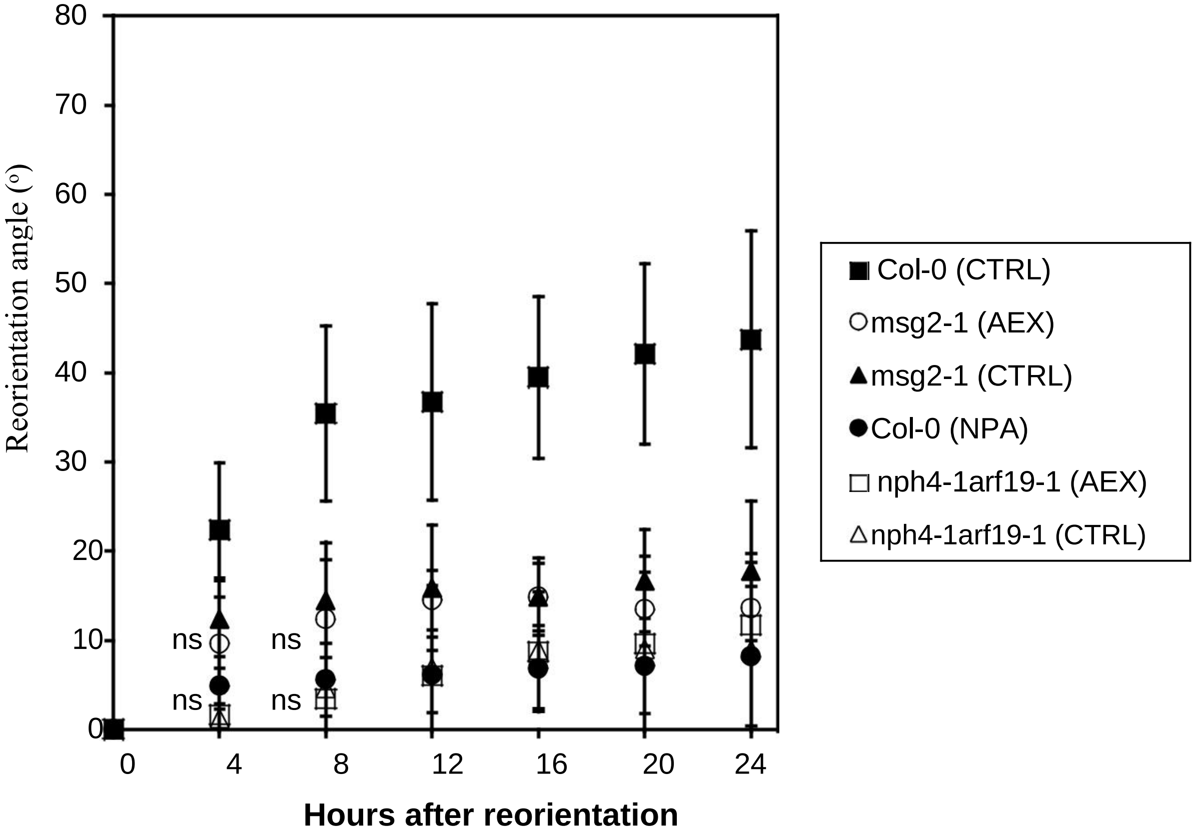

Supplementary Fig. S7 [^{3}H]NAA Accumulation Kinetics in Tobacco BY-2 Cells Upon 100 µM AEX or/and 100 µM ACC Treatments. All treatments contained 0.1% DMSO. Time of AEX/ACC Addition is Shown by the Arrow. Error bars indicate SD (*n*=4).

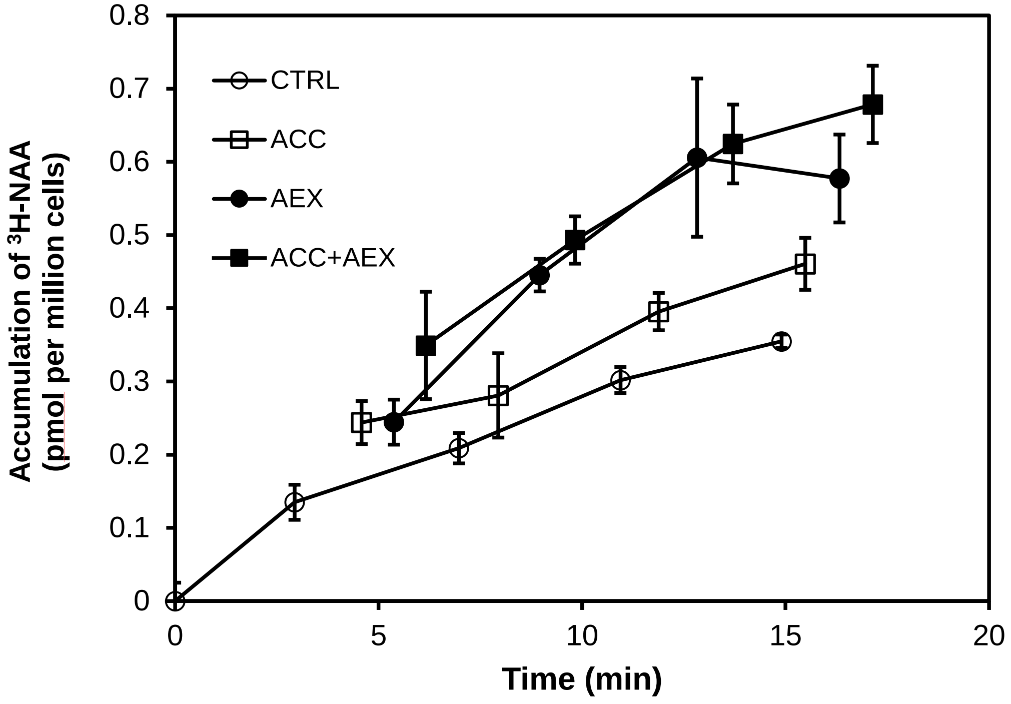

Supplementary Fig. S8 GC-MS/MS Determination of the Endogenous Content of IAA, IAA Catabolites and IAA Conjugates of four-day Etiolated Seedlings Treated with AEX. IAA and 2-Oxindole-3-Acetic Acid (oxIAA) and oxIAA-glucose ester (oxIAA-GE) content in **(A)** Cotyledons (including Shoot Apical Meristems) and **(B)** Hypocotyls; **(C)** Endogenous IAA or IAA Conjugate Contents in Cotyledons (including SAMs) and Hypocotyls. Contents are shown for Col-0 treated with 0.05% DMSO (CTRL), 10 µM ACC and 50 µM AEX. All treatments contained 0.05% DMSO. Error bars indicate SD (n=2~5).

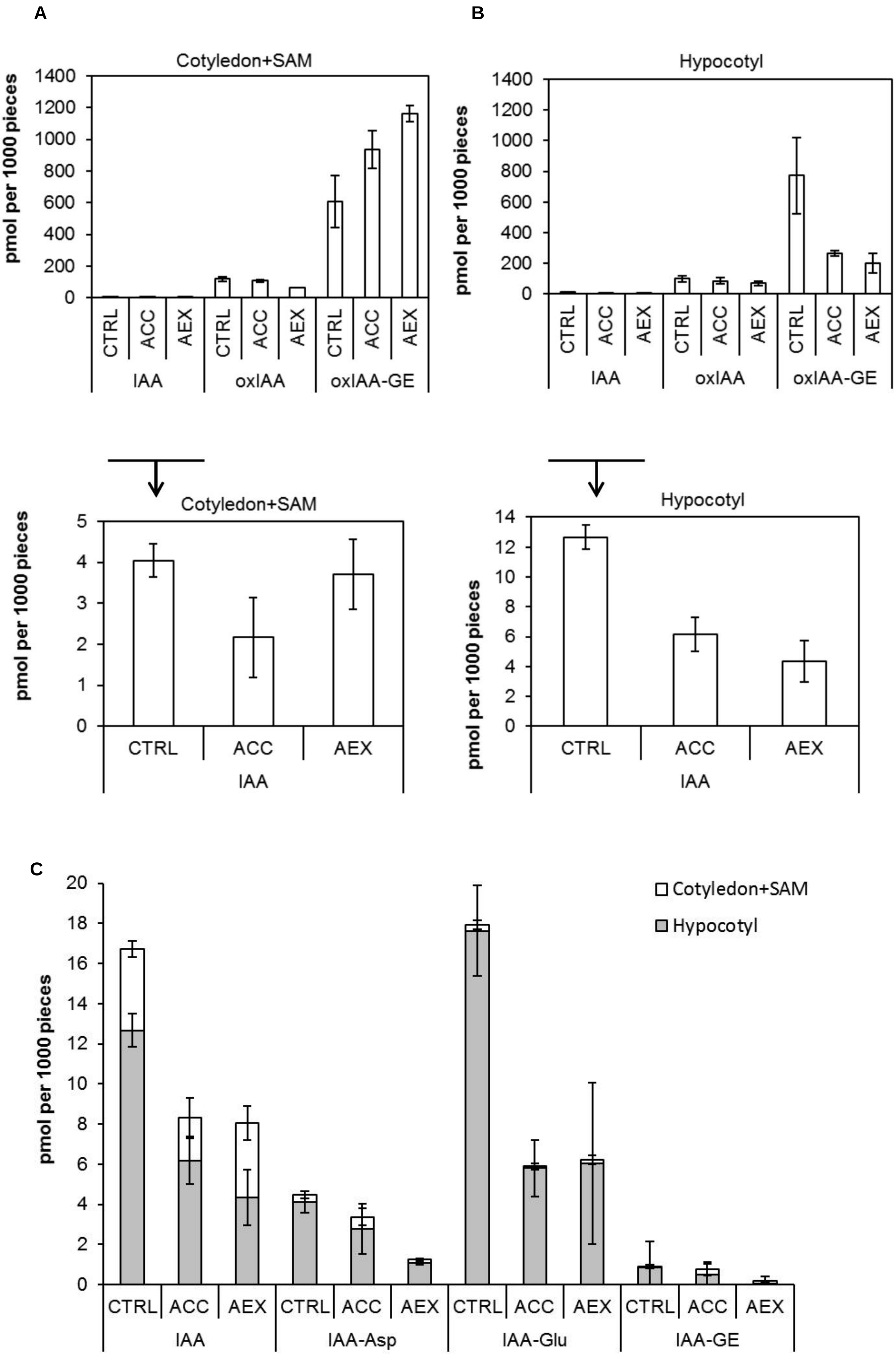

A
B
Cotyledon+SAM
Hypocotyl
pmol per 1000 pieces
CTRL
ACC
AEX
IAA
oxIAA
oxIAA-GE
C
Cotyledon+SAM
Hypocotyl
IAA-Asp
IAA-Glu
IAA-GE

Supplementary Fig. S9 Effects of AEX and its analogs on phenotypes of etiolated seedlings and on the gravitropic response of hypocotyls. (A) Phenotypic effects of AEX and its analogs at 10 μM and 50 μM on four-day dark-grown seedlings. Chemical structures of analogs are listed in Supplementary Table S6. The individual photographs were cropped without changing the scale; the black background was post-added. Scale bar = 1 mm. **(B)** Reorientation assay on three-day-old dark-grown seedlings (two days after germination, see details in legend of Figure 7). Col-0 were grown in the presence of AEX (50 μM) or E (10 μM) or D (1 μM) or mock treated (DMSO). The reorientation kinetics in the presence of 10 μM of E or 1 μM of D revealed an enhanced rate of reorientation visible at 12 hours. Data are presented as mean ± SD**.**

A

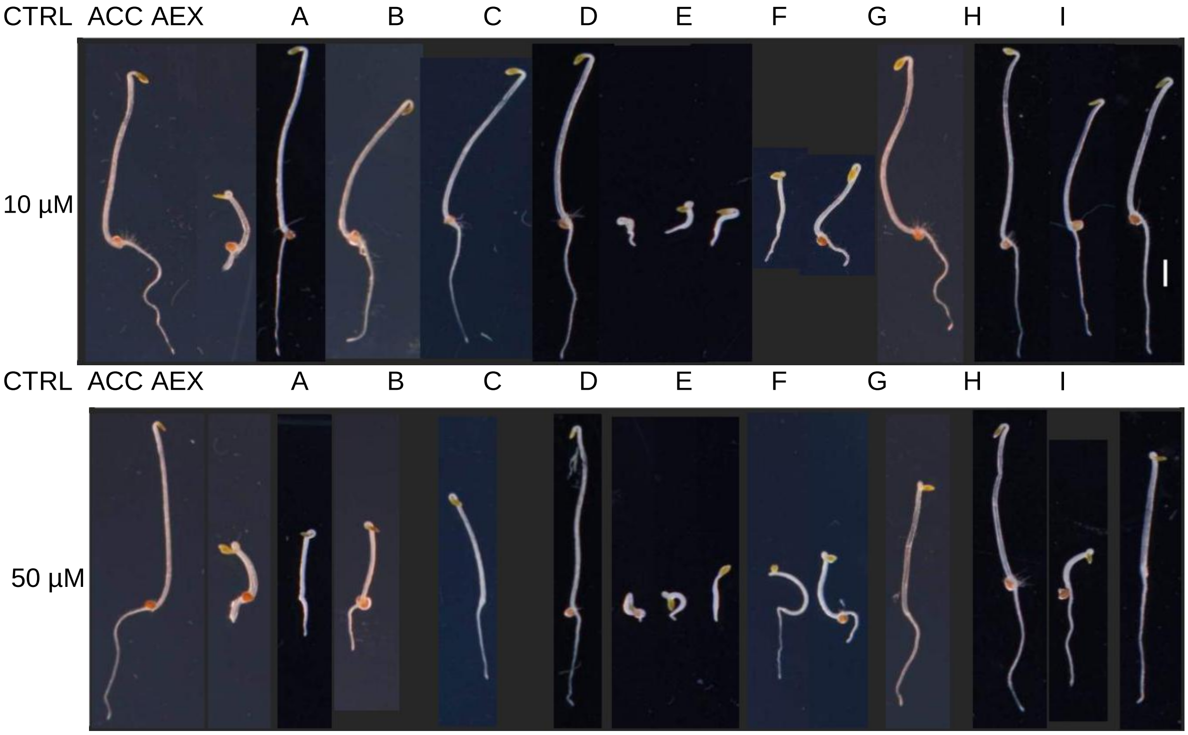
CTRL ACC AEX A B C D E F G H I
10 µM
CTRL ACC AEX A B C D E F G H I
50 µM

B

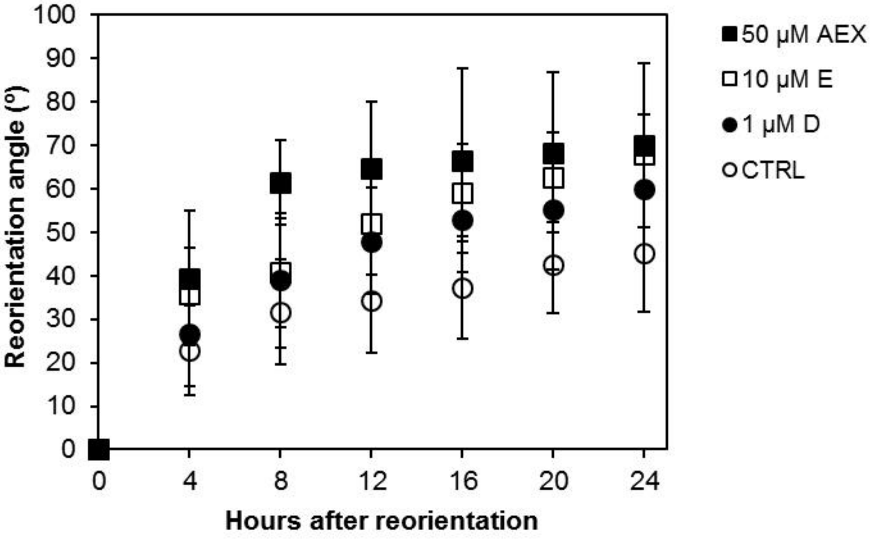
Reorientation angle (°)
Hours after reorientation
0
10
20
30
40
50
60
70
80
90
100
0
4
8
12
16
20
24
50 µM AEX
10 µM E
1 µM D
CTRL

Supplementary Fig. S10 Phenotype of Light-Grown Plants. Plants were grown on 1/2 MS medium containing 1% sucrose in the presence of 0.05% DMSO (CTRL), 10 µM ACC, 50 µM AEX. All treatments contained 0.05% DMSO. Photographs were taken at day seven, 21, 35 and 42. Scale bar = 1 cm.

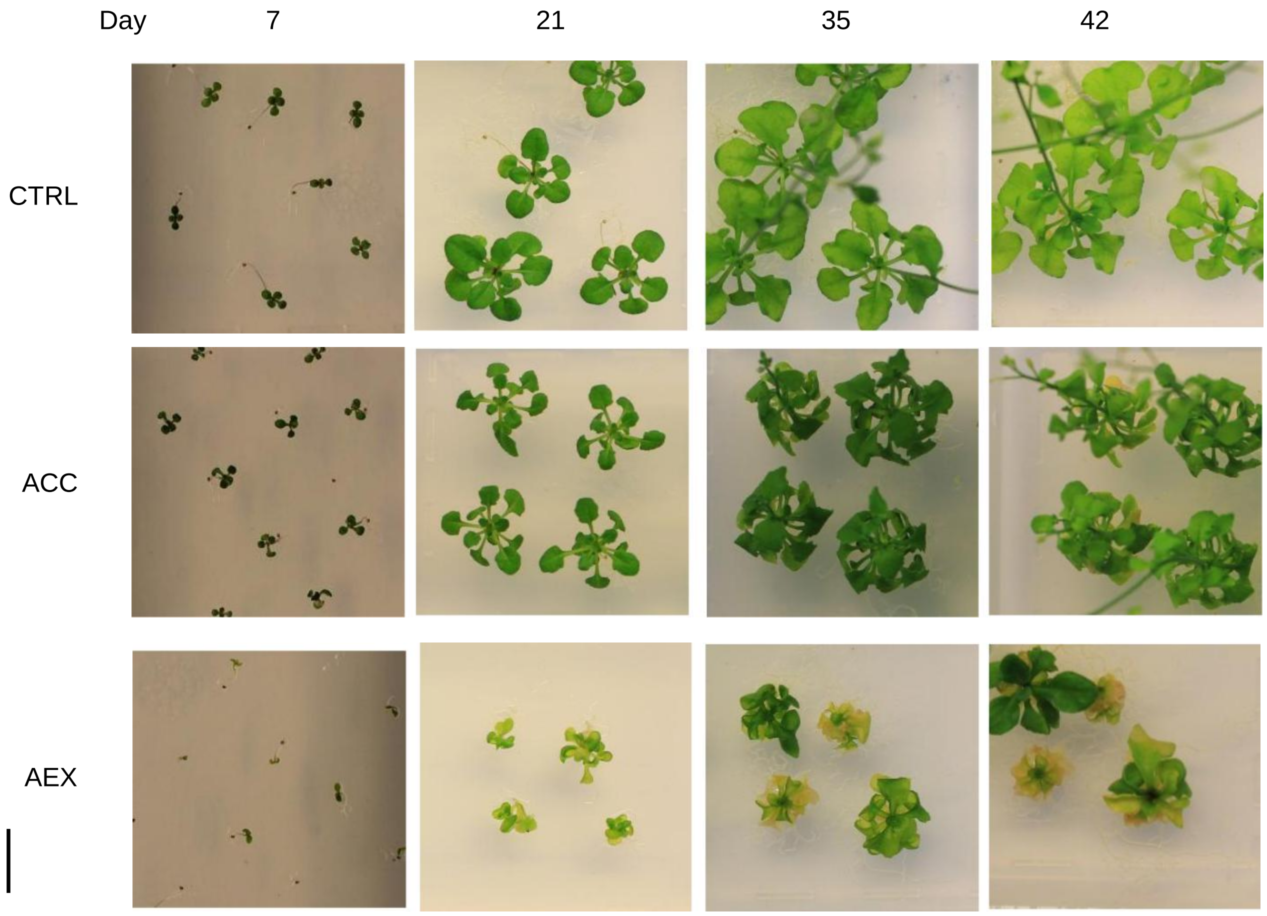

Supplementary Fig. S11 *In silico* docking simulation of IAA and AEX. Representation of binding cavity of TIR1 (presented as surface model) with the predicted position of (**A**) IAA (blue stick model; binding affinity = -7.6 kcal/mol) and (**B**) AEX (yellow stick model; -5.9 kcal/mol) as determined in Autodock Vina. Red stick model in (**A-B**) represents the position of IAA determined experimentally through X-ray diffraction. For clarity the IAA7 protein was masked in the figure, although it was included in the docking analysis.

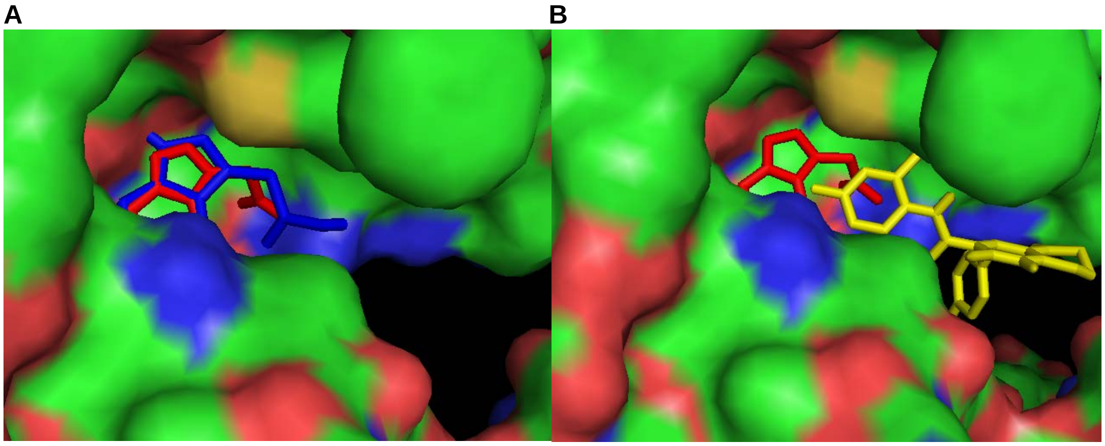

Supplementary Table S1 Summary of statistical analysis. (See Supplementary Tables S1-S5.xlsx)

Supplementary Table S2 Genes regulated by 6 hours AEX treatment. (See Supplementary Tables S1-S5.xlsx)

Supplementary Table S3 Gene Ontology of AEX regulated genes. (See Supplementary Tables S1-S5.xlsx)

Supplementary Table S4 Common genes regulated by AEX and other arrays. (See Supplementary Tables S1-S5.xlsx)

Supplementary Table S5 Cell wall related genes which expression decreased by minimal 4-fold after 6 hours AEX treatment. (See Supplementary Tables S1-S5.xlsx)

Supplementary Table S6 The minimal tested concentration (10 or 50 μM) of AEX analogs to induce the apical
hook curvature**.**

Name	Chembridge ID	Chemical Structures	IUPAC	Conc. (μM)*
AEX	6527749	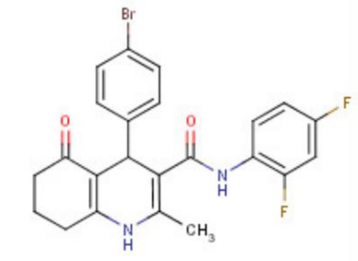	4-(4-bromophenyl)-N-(2,4-difluorophenyl)-2-methyl-5-oxo-1,4,5,6,7,8-hexahydro-3-quinolinecarboxamide	50
A	6514196	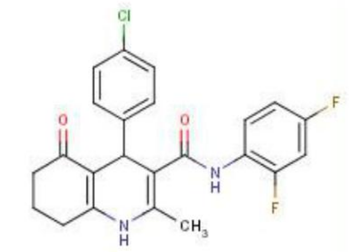	4-(4-chlorophenyl)-N-(2,4-difluorophenyl)-2-methyl-5-oxo-1,4,5,6,7,8-hexahydro-3-quinolinecarboxamide	50
B	6520852	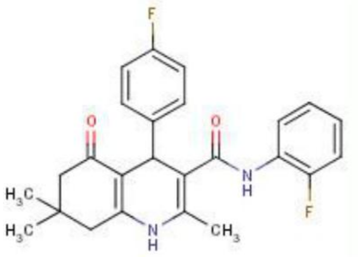	N-(2-fluorophenyl)-4-(4-fluorophenyl)-2,7,7-trimethyl-5-oxo-1,4,5,6,7,8-hexahydro-3-quinolinecarboxamide	50
C	6640029	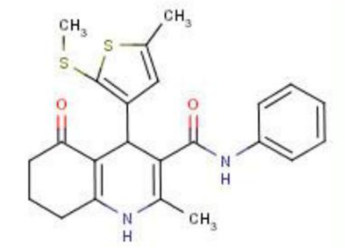	2-methyl-4-[5-methyl-2-(methylthio)-3-thienyl]-5-oxo-N-phenyl-1,4,5,6,7,8-hexahydro-3-quinolinecarboxamide	N/A
D	5754347	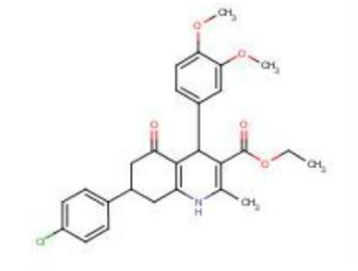	ethyl 7-(4-chlorophenyl)-4-(3,4-dimethoxyphenyl)-2-methyl-5-oxo-1,4,5,6,7,8-hexahydro-3-quinolinecarboxylate	10

E	5712036 (LATCA)	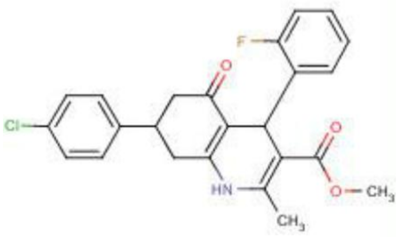	methyl 7-(4-chlorophenyl)-4-(2-fluorophenyl)-2-methyl-5-oxo-1,4,5,6,7,8-hexahydro-3-quinolinecarboxylate	10
F	5473152 (LATCA)	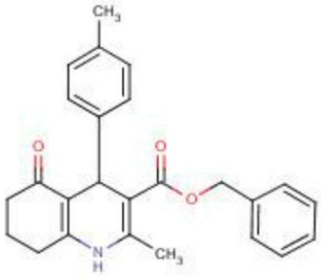	benzyl 2-methyl-4-(4-methylphenyl)-5-oxo-1,4,5,6,7,8-hexahydro-3-quinolinecarboxylate	50
G	5707885 (LATCA)	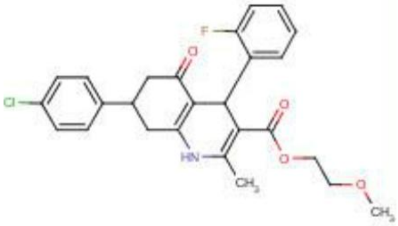	2-methoxyethyl 7-(4-chlorophenyl)-4-(2-fluorophenyl)-2-methyl-5-oxo-1,4,5,6,7,8-hexahydro-3-quinolinecarboxylate	N/A
H	5617132 (LATCA)	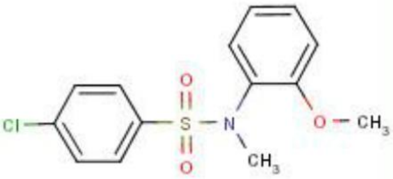	4-chloro-N-(2-methoxyphenyl)-N-methylbenzenesulfonamide	50
I	5601004 (LATCA)	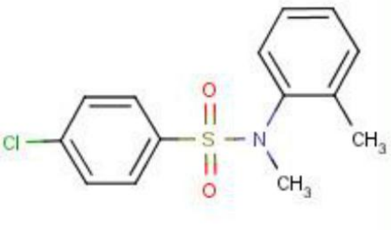	4-chloro-N-methyl-N-(2-methylphenyl)benzenesulfonamide	50

*: The minimal tested concentration (10 or 50 µM) which can induce the apical hook curvature compared to the Col-0 mock treated control
N/A: no effect detected

Supplementary Protocols S1. ACCERBATIN (AEX) stability in vivo determined by liquid chromatography– mass spectrometry (LC-MS) profiling

ACCERBATIN (AEX) stability in vivo determined by liquid chromatography–mass spectrometry (LC-MS) profiling

All chromatograms were processed, integrated and aligned as published before (Morreel *et al.*, 2014). In total, this yielded 5692 m/z features that could be putatively assigned to 822 compounds following the "peak grouping" algorithm previously described (Morreel *et al.*, 2014). Besides the AEX compound itself, only one other "peak group" (called AEXfrg) was observed to be solely present in those samples that were fed with the AEX compound. Following MS data for AEX and AEXfrg were obtained (relative abundance versus the base peak is given between parentheses for each product ion):

Compound AEX. m/z 471.05251 $[M\text{-}H^+]^-$ ($C_{23}H_{18}O_2N_2{}^{79}BrF_2{}^-$, ppm = -0.019). MS^2 (collision energy 35%): 295 (45), 315 (100), 451 (79). MS^3 of first product ion at m/z 315 (collision energy 35%): 295 (100). MS^3 of first product ion at m/z 451 (collision energy 35%): 295 (3), 369 (1), 407 (2), 423 (22), 433 (29), 451 (100). MS^4 of second product ion at m/z 295 derived from the first product ion at m/z 315 (collision energy 35%): 223 (1), 253 (2), 259 (1), 267 (2), 275 (9), 277 (15), 280 (2), 293 (1), 295 (100).

Compound AEXfrg. m/z 370.04467 $[M\text{-}H^+]^-$ ($C_{19}H_{17}O_2N_1{}^{79}Br^-$, ppm = -0.039). MS^2 (collision energy 35%): 196 (2), 212 (1), 214 (100), 342 (1), 370 (64). MS^3 of first product ion at m/z 214 (collision energy 35%): 134 (3), 160 (5), 172 (7), 178 (3), 186 (21), 196 (100), 199 (16). MS^4 of second product ion at m/z 196 (collision energy 35%): 178 (100).

The loss of both fluorines and one nitrogen in the chemical formulae of AEXfrg as compared to that of AEX indicates that AEXfrg is formed via cleavage of the amide bond in AEX. However, such a cleavage would also have resulted in the loss of 6 carbons and 4 hydrogens which cannot be deduced by comparing both chemical formulae. Clearly, the reaction proceeded with the addition of an ethylene moiety.

Supplementary Protocols S2. AEX stability in vitro determined by Nuclear magnetic resonance (NMR)

Given the structure of the component of interest (AEX) with brutoformula $C_{23}H_{19}BrF_2N_2O_2$, it was noticed that after heating a certain amount of product degraded to $C_{19}H_{17}O_2NBr$, which corresponds to a loss of a $C_4H_2NF_2$ – fragment. This suggests that at least a part of the fluorinated aromatic ring is fragmented during the heating step. The aim of the NMR measurements discussed here is to explore whether the same fragmentation event can be reproduced and in the case this happens if the new compound after fragmentation can be identified.

Regarding the spectra shown in the this paragraph, a remark has to be made: considering the fact that the original samples were already dissolved in 53 µl of protonated methanol, the baseline of several spectra will be distorted due to the intensity of the two methanol signals. In addition, overlap of the previously mentioned signals with some signals of the molecule of interest cannot be excluded: as can be seen in Figure 2, all the resonances can be assigned except for proton n° 14, which is expected to be located around 4.7 ppm (chemical shift prediction ChemDraw Ultra 13, numbering corresponds with the numbering in Figure 1) and is believed to be overlapping with the second very intense methanol signal residing at 4.80 ppm.

The assignment of the molecule itself is fairly straightforward and can be performed almost solely on the basis of chemical shift and integral values, except for the amine n°10 and amide n°8 where none of the two can be assigned unambiguously. In addition, as can be seen in Figure 2, the integral values are in agreement with the number of protons assigned to each ^1H signal. It has to be noted that when the signal is situated closer to one of the methanol signals, the integral will start to deviate from the correct value due to the partial overlap of the large background of these solvent signals with the signal of interest (e.g. n°13 should correspond with 2 protons, where the integral corresponds with 2.8). In addition, it can be noted that some small impurities are present as well.

Regarding the first comparison of the three different samples in Figure 3, measured at room temperature under identical conditions, it can be noted that there are no significant differences in the signals of interest: both sets of aromatic signals are present and no new signals compared to signals of the AEX component are visible.

During the temperature study, several 1D ^1H measurements were performed at regular intervals of ±30min. Figure 4 shows three spectra, one at the start of the temperature study and two spectra measured after 6 hours and 12 hours of heating at 50°C. As can be seen clearly, no changes

whatsoever can be noted throughout the experiments. In special interest, highlighted in the three spectra, the aromatic signals remain identical.

As a last type of experiment, two samples were subjected to a pH study: both the reference sample which wasn't heated as the sample prior heated at 80°C, were both measured at pH ±5 and ±4. As can be seen from Figure 5 and 6, no notable differences concerning the signals of interest as any new signals can be observed.

Considering both the temperature as the pH study don't show any notable differences before and after heating/pH adjustment, one has to conclude the AEX compound is both thermal and pH stable. Another possibility could be that the fragmentation is indeed happening but the resulting fragment and changes in the signals of interest are below the NMR detection limit.

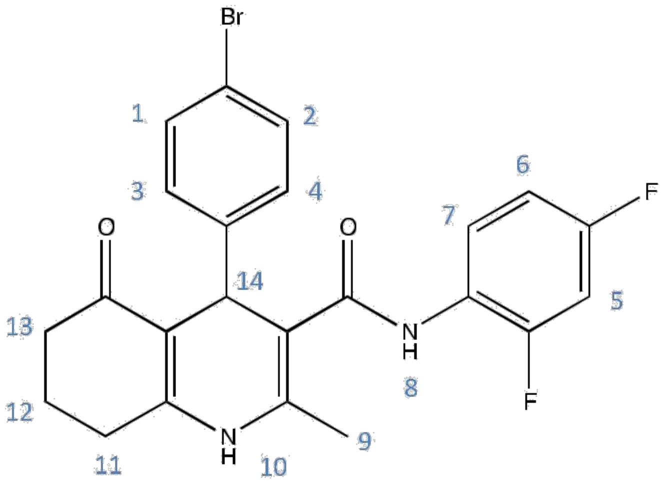

Figure 1 overview general structure and numbering used throughout the NMR assignment procedure.

Overview 1D 1H assignment

δ 1H(ppm)	multiplicity	Integral	# protons	Annotation
1,9/2,0	multiplet	1,13; 0,87	2	12
2,15	singlet	3,26	3	9
2,31	multiplet	2,37	2	11
2,54	multiplet	2,82	2	13
6,88	triplet	0,86	1	5
6,97	triplet of doublets	0,89	1	6
7,2	doublet	2,02	2	3 & 4
7,36	doublet	2	2	1 & 2
7,45	multiplet	0,89	1	7
8,75	broad singlet	0,61	1	8 or 10
9,12	broad singlet	0,64	1	8 or 10
		**Total**	18	

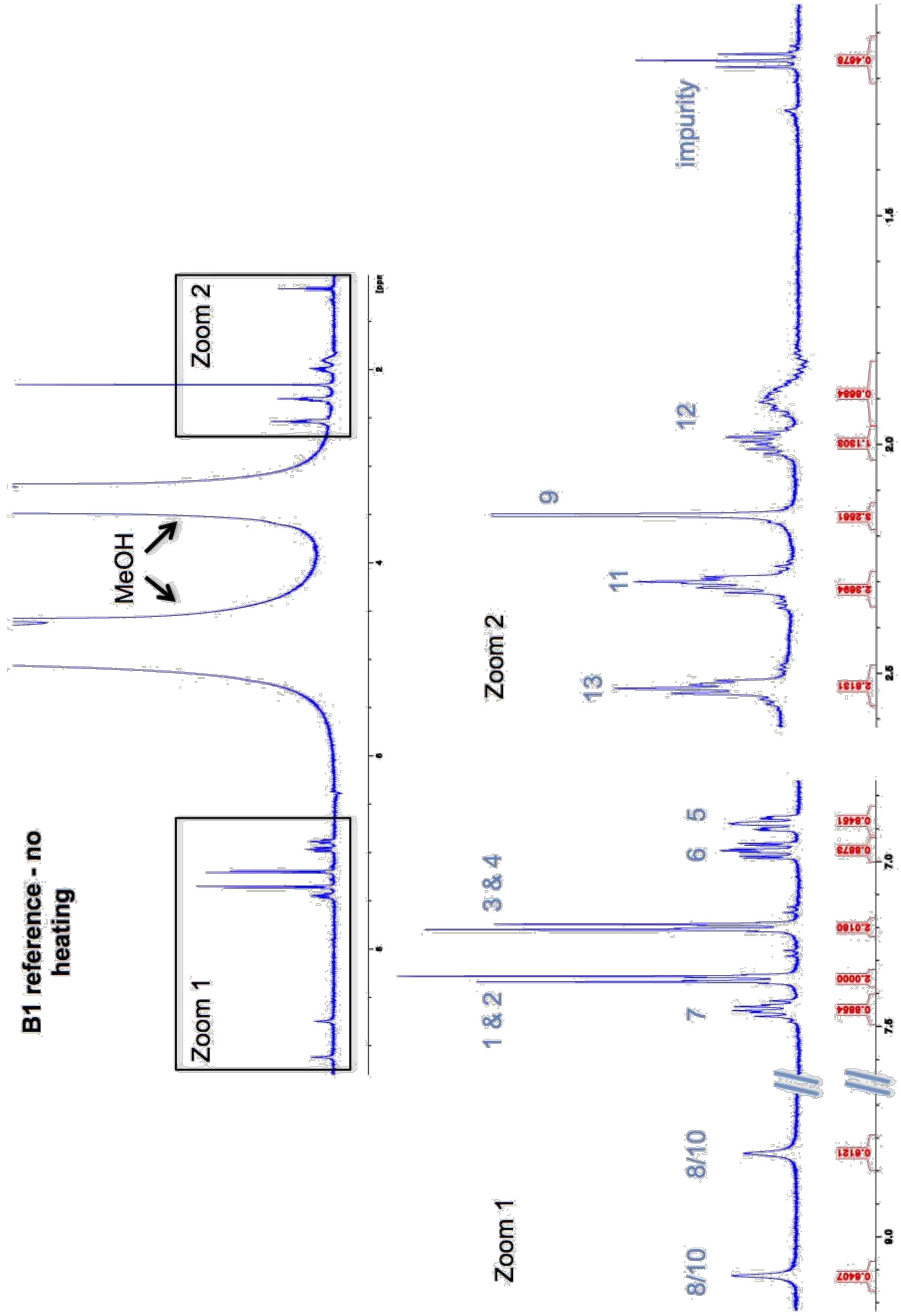

Figure 2: Overview general 1D 1H assignment of the AEX component (25°C, 500MHz).

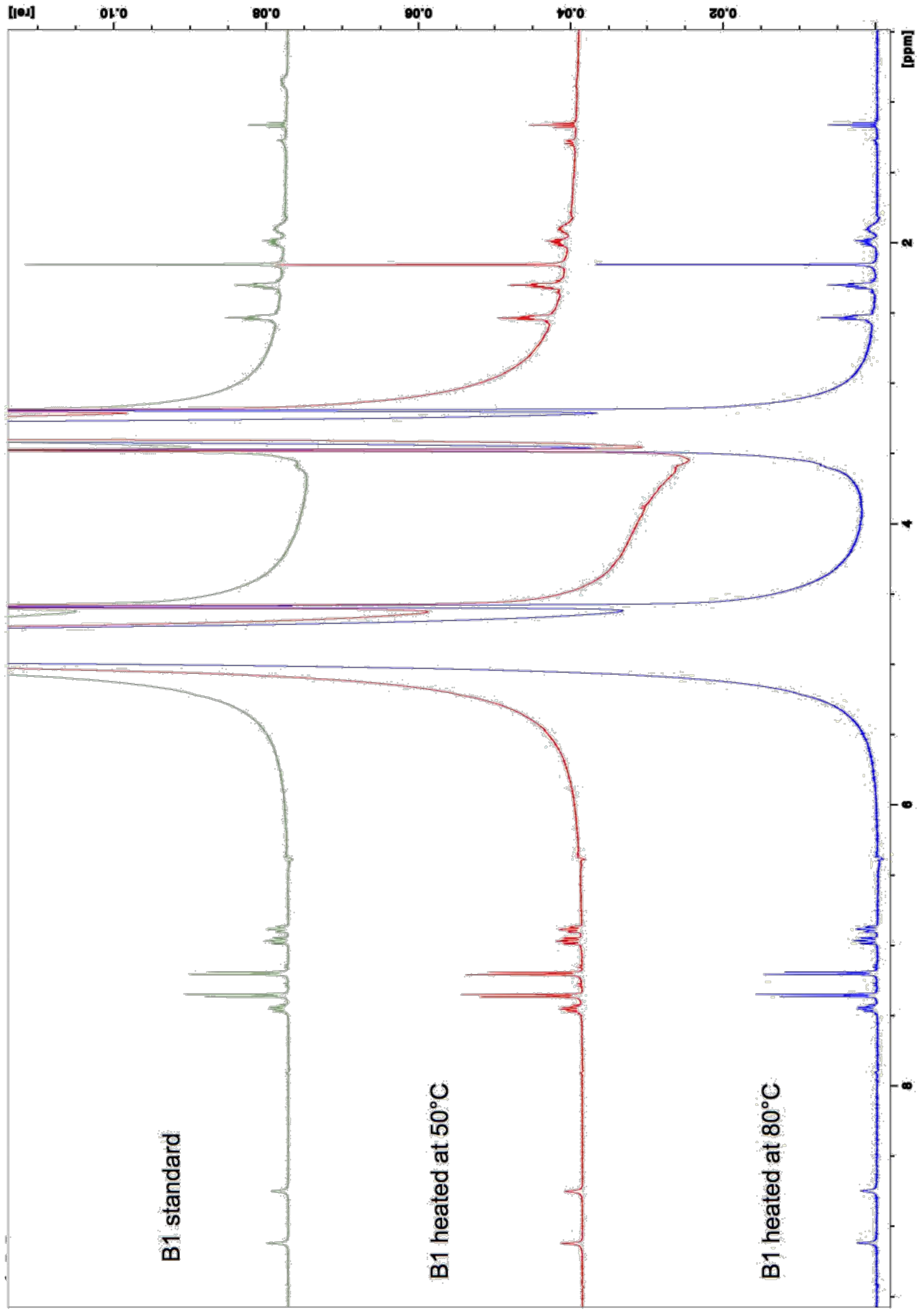

Figure 3: Overview of the three samples measured at room temperature (25°C, 500MHz).

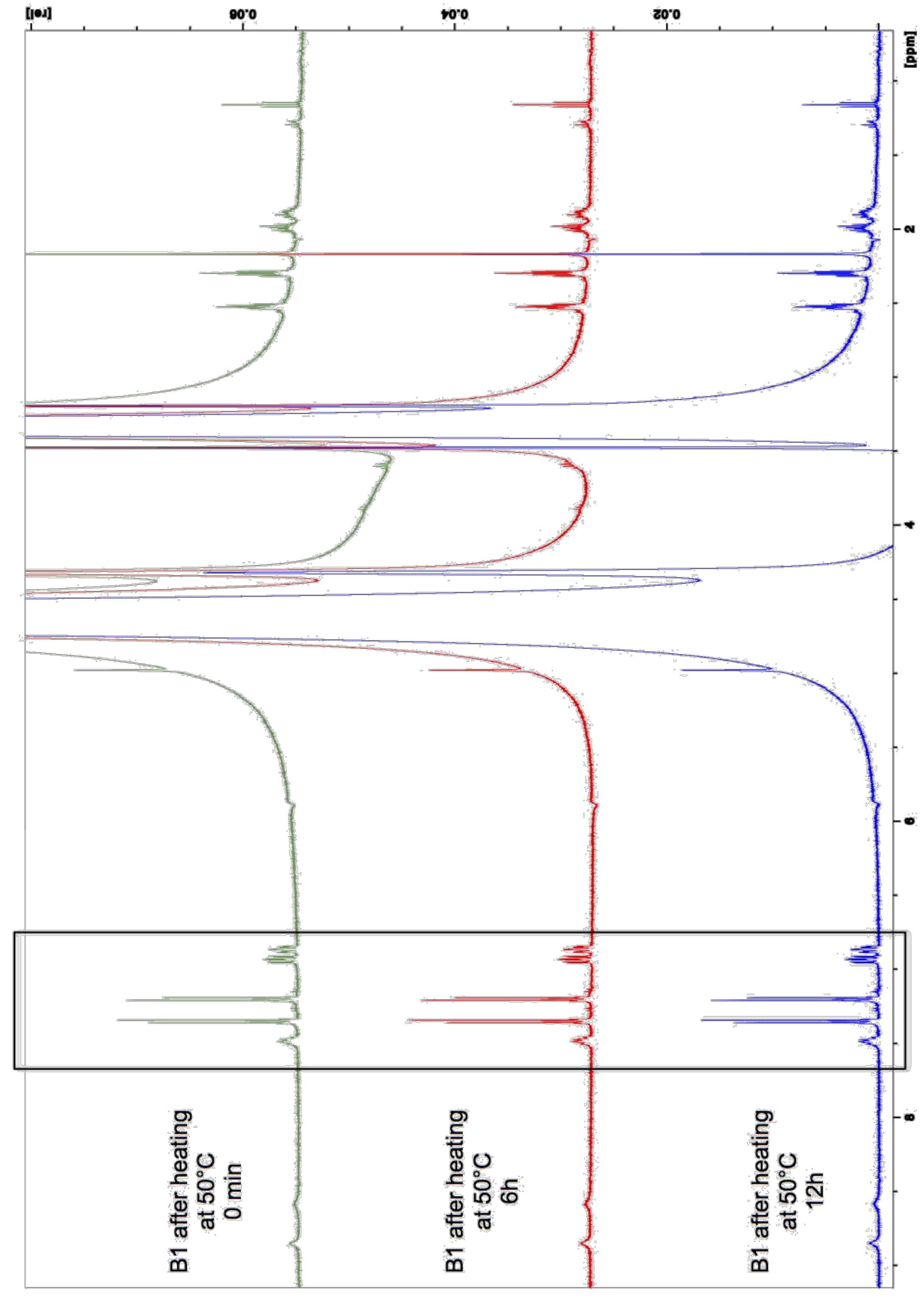

Figure 4: Overview of the AEX component temperature study at 50°C after 0, 6 and 12 hours time (500MHz).

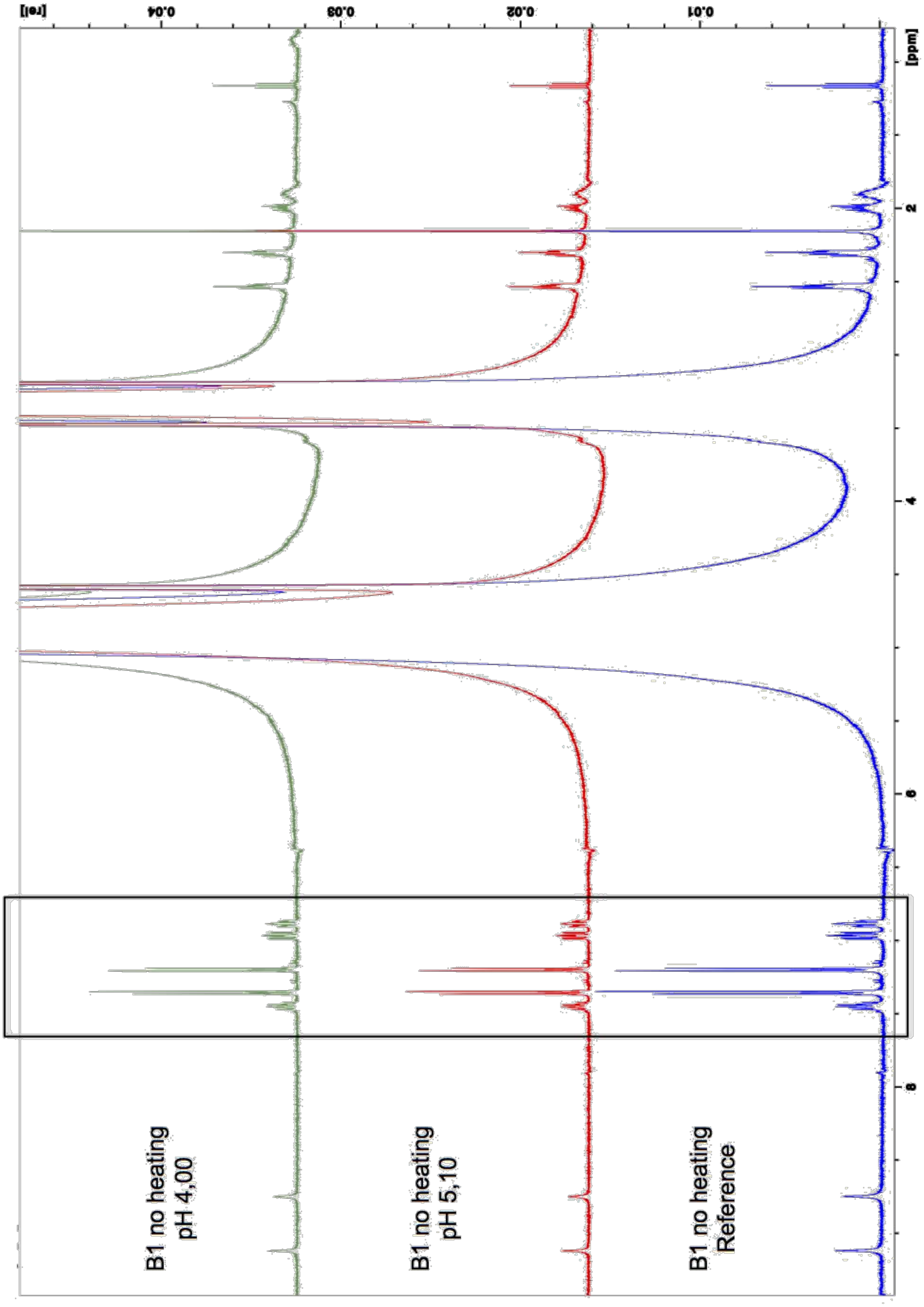

Figure 5: Overview pH study of the AEX reference component (25°C, 500MHz).

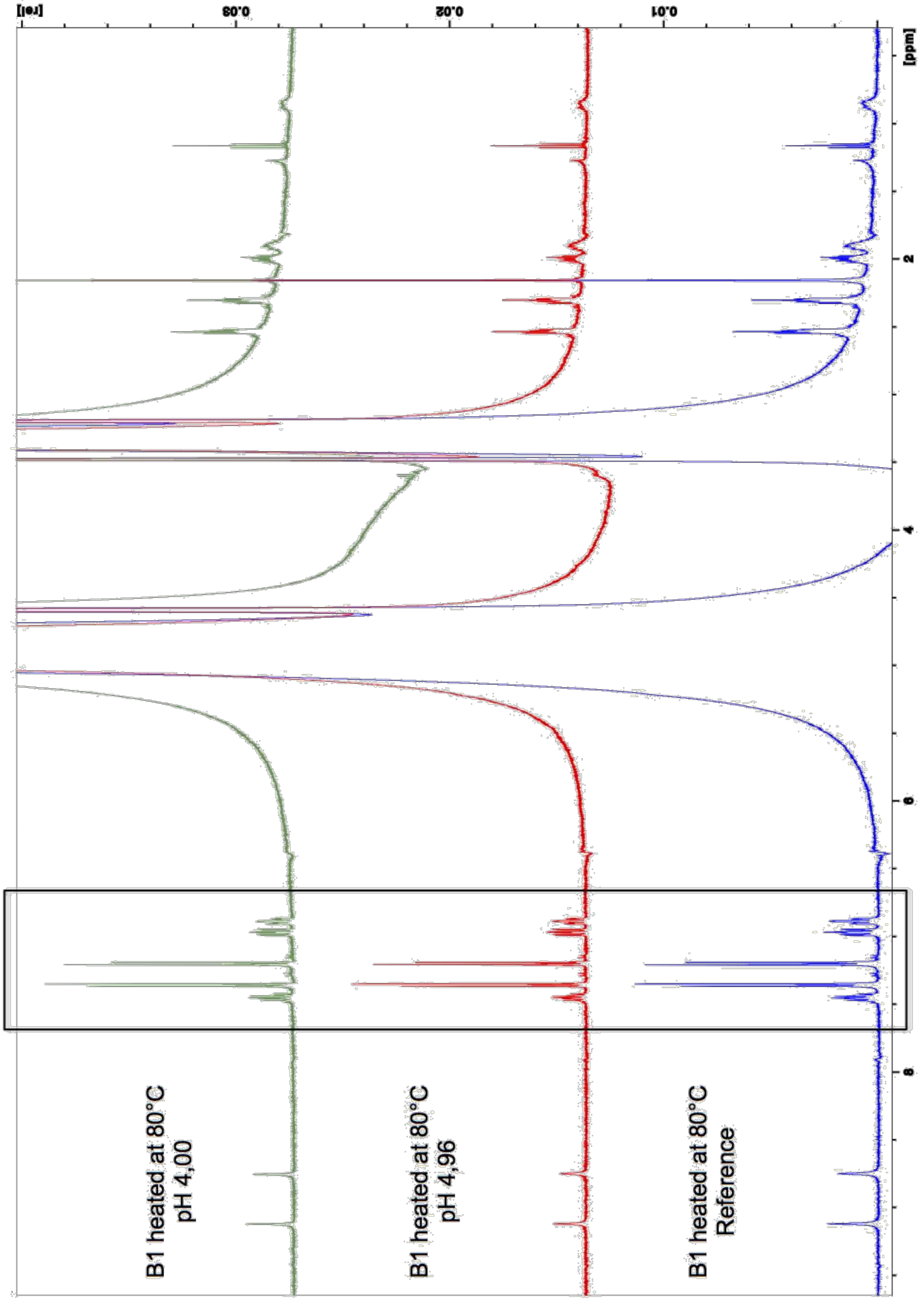

Figure 6: Overview pH study of the AEX component prior heated at 80°C (25°C, 500MHz).

Supplementary Protocols S3. Kinematic and genetic analysis of the effect of AEX on hook development

The exaggerated apical hook curvature is one of the key features of AEX (Fig. 1b, Supplementary Fig. S4A). To know when AEX starts to act in hook development, a kinematic analysis was performed. The apical hook of etiolated Col-0 seedlings displayed three constitutive phases of development, consistent with previous results: the formation (0~36 hours), maintenance (36~48 hours) and opening phase (48~144 hours) (Supporting Information Fig.S3B) (Vandenbussche *et al*., 2010; Smet *et al*., 2014). ACC-treated wild-type seedlings exhibited a significant hook exaggeration (~235° during maintenance phase), and were characterized by an extended formation phase (0~48 hours) and prolonged opening phase (252 hours after germination), while the rate of opening was similar to control. AEX-treated Col-0 seedlings also showed three distinct phases of hook development, but exhibited a formation phase lasting 3.5 days and a prolonged maintenance phase (84~132 hours; 258°) compared to control seedlings. The hook opening rate was slower, and not even completed after 360 hours post germination. Finally, when AEX and ACC were combined, effects on all three phases of apical hook development were even more pronounced. Hence, the combined treatment led to a significantly more exaggerated curvature than that of AEX- or ACC-treated seedlings.

Hook development in *Arabidopsis* is strongly controlled by the HOOKLESS 1 (HLS1) gene (An *et al*., 2012). Kinetic analysis of *hls1-1* hook development revealed that both control and ACC-treated seedlings immediately entered the opening phase and reached an angle of 0° at 48 hours after germination (Supplementary Fig. S4A and C). Upon AEX treatment however, *hls1-1* seedlings formed a conspicuous hook structure (until 132° at 24 hours after germination), and subsequently started opening, which was completed at 90 hours after germination. Thus, AEX is likely acting downstream of HLS1.

As auxin is involved downstream of ethylene signaling and also acts in parallel to the ethylene pathway (Stepanova *et al*., 2007), selected auxin signaling (*arf2-6; nph4-1arf19-1; axr3-1* (Okushima *et al*., 2005; Vandenbussche *et al*., 2010; Zadnikova *et al*., 2010)) and transport mutants (*aux(s)lax3; aux1-7; 35S::PIN1; pin3-3; rcn1-1; pid(s); wag(s); pgp4-1; abcb1abcb19* (Friml *et al*., 2002; Vandenbussche *et al*., 2010; Wu *et al*., 2010; Zadnikova *et al*., 2010; Chen *et al*., 2013)) were screened in the presence of 50 µM AEX (Supplementary Fig. S5A). Apical hook development was enhanced by AEX in all lines. In the presence of exogenous auxin (IAA or 2,4-

D) or the auxin efflux inhibitor N-(1-naphtyl)phtalamic acid (NPA), no hook was observed at day 4 in seedlings treated with AEX (Supplementary Fig. S5B). In contrast, a hook was still seen when 1-naphthoxyacetic acid (1-NOA), an auxin influx inhibitor, was applied. Thus, AEX required a threshold level of auxin to induce a full response of apical hook curvature, as seen in Col-0.

Supplementary References

An F, Zhang X, Zhu Z, Ji Y, He W, Jiang Z, Li M, Guo H. 2012. Coordinated regulation of apical hook development by gibberellins and ethylene in etiolated *Arabidopsis* seedlings. *Cell Res*.

Chen IJ, Lo WS, Chuang JY, Cheuh CM, Fan YS, Lin LC, Wu SJ, Wang LC. 2013. A chemical genetics approach reveals a role of brassinolide and cellulose synthase in hypocotyl elongation of etiolated *Arabidopsis* seedlings. *Plant Sci* **209**: 46-57.

Friml J, Wisniewska J, Benkova E, Mendgen K, Palme K. 2002. Lateral relocation of auxin efflux regulator PIN3 mediates tropism in *Arabidopsis*. *Nature* **415(6873):** 806-809.

Morreel K, Saeys Y, Dima O, Lu F, Van de Peer Y, Vanholme R, Ralph J, Vanholme B, Boerjan W. 2014. Systematic Structural Characterization of Metabolites in *Arabidopsis* via Candidate Substrate-Product Pair Networks. *Plant Cell* **26**:929-945.

Vandenbussche F, Petrasek J, Zadnikova P, Hoyerova K, Pesek B, Raz V, Swarup R, Bennett M, Zazimalova E, Benkova E, *et al*. 2010. The auxin influx carriers AUX1 and LAX3 are involved in auxin-ethylene interactions during apical hook development in *Arabidopsis thaliana* seedlings. *Development* **137(4):** 597-606.

Wu G, Cameron JN, Ljung K, Spalding EP. 2010. A role for ABCB19-mediated polar auxin transport in seedling photomorphogenesis mediated by cryptochrome 1 and phytochrome B. *Plant J* **62(2):** 179-191.

Zadnikova P, Petrasek J, Marhavy P, Raz V, Vandenbussche F, Ding ZJ, Schwarzerova K, Morita MT, Tasaka M, Hejatko J, *et al*. 2010. Role of PIN-mediated auxin efflux in apical hook development of *Arabidopsis thaliana*. *Development* **137(4):** 607-617.
